# Cortical cells reveal APP as a new player in the regulation of GABAergic neurotransmission

**DOI:** 10.1038/s41598-017-00325-2

**Published:** 2017-03-23

**Authors:** Anna Doshina, Florian Gourgue, Michiho Onizuka, Remi Opsomer, Peng Wang, Kunie Ando, Bernadette Tasiaux, Ilse Dewachter, Pascal Kienlen-Campard, Jean-Pierre Brion, Philippe Gailly, Jean-Noël Octave, Nathalie Pierrot

**Affiliations:** 1 Institute of Neuroscience, Université catholique de Louvain, 1200 Brussels, Belgium; 2 Laboratory of Histology and Neuropathology, Université libre de Bruxelles, 1070 Brussels, Belgium

## Abstract

The amyloid precursor protein (APP) modulates synaptic activity, resulting from the fine tuning of excitatory and inhibitory neurotransmission. GABAergic inhibitory neurotransmission is affected by modifications in intracellular chloride concentrations regulated by Na^+^-K^+^-2Cl^−^ cotransporter 1 (NKCC1) and neuronal K^+^-Cl^−^ cotransporter 2 (KCC2), allowing entrance and efflux of chloride, respectively. Modifications in NKCC1 and KCC2 expression during maturation of cortical cells induce a shift in GABAergic signaling. Here, we demonstrated that APP affects this GABA shift. Expression of APP in cortical cells decreased the expression of KCC2, without modifying NKCC1, eliciting a less inhibitory GABA response. Downregulation of KCC2 expression by APP was independent of the APP intracellular domain, but correlated with decreased expression of upstream stimulating factor 1 (USF1), a potent regulator of *Slc12a5* gene expression (encoding KCC2). KCC2 was also downregulated *in vivo* following APP expression in neonatal mouse brain. These results argue for a key role of APP in the regulation of GABAergic neurotransmission.

## Introduction

The amyloid precursor protein (APP) is critically involved in the pathophysiology of Alzheimer’s disease (AD)^1^, the most prevalent cause of dementia affecting more than 44 million people worldwide (from World Alzheimer Report 2015). Accumulation of amyloid beta peptide (Aβ), derived from the proteolytic cleavage of APP^2^, is one of the main hallmarks of AD^3, 4^. Consecutive cleavage of APP by two membrane-bound aspartic proteases termed β- and γ-secretases not only leads to the production of Aβ but also generates the APP intracellular domain (AICD), a physiologically active catabolite that could control the transcription of several genes^5^. Besides producing Aβ, many physiological functions of APP have been described^6, 7^. Amongst these, APP and/or its catabolites were shown to influence synaptic excitatory^8, 9^ and inhibitory^10–14^ neurotransmission, both *in vitro* and *in vivo* ^15^.

In the adult central nervous system, γ-amino-butyric acid (GABA) is a major inhibitory neurotransmitter that shapes cortical activity^16^. Interplay between excitation and inhibition ensures the stability of cortical activity by preventing runaway excitation^17, 18^ and regulates behavioral functions, such as learning and memory, supported by the long-term potentiation (LTP) mechanism^16, 19^. GABA-mediated inhibition involves both conductance-based (shunting) and voltage-based (hyperpolarization) mechanisms^20^. Even though GABA is mainly hyperpolarizing and inhibitory in the mature brain, its actions are primarily depolarizing and sometimes excitatory in the developing brain^21–24^. GABAergic neurotransmission is mediated by two main types of receptors: metabotropic GABA_B_ receptors (GABA_B_R) and ionotropic GABA_A_ receptors (GABA_A_R). Since GABA_A_R are ligand-gated ion channels that mainly allow the bidirectional flux of Cl^−^ ions, the nature of GABAergic neurotransmission, depolarizing versus hyperpolarizing, is essentially determined by intracellular concentrations of Cl^−^ ([Cl^−^]_i_) and the membrane potential.

The two main cation-chloride cotransporters regulating the [Cl^−^]_i_ in the central nervous system (CNS) are Na^+^-K^+^-2Cl^−^ cotransporter 1 (NKCC1) allowing the entrance of Cl^−^ and K^+^-Cl^−^ cotransporter 2 (KCC2), allowing the efflux of chloride^20, 25, 26^. Changes in NKCC1 and KCC2 expression levels influence the polarity of inhibitory currents mediated by GABA or glycine, another inhibitory neurotransmitter. For instance, increased KCC2 expression levels lead to an increase in Cl^−^ extrusion, a reduction in [Cl^-^]_i_ and subsequent conversion of inhibitory neurotransmission from depolarizing to hyperpolarizing^27^.

In APP-deficient mice, attenuation of GABA-mediated inhibitory post-synaptic currents was shown to contribute to the impairment of synaptic plasticity illustrated by LTP and behavioural deficits^10, 11^. Theta-gamma oscillation coupling, involved in inhibitory transmission^28^, was also demonstrated to be impaired in these mice^13^. Moreover, APP overexpression, but not a subsequent Aβ increase, leads to hypersynchronous network activity in an APP transgenic mouse model of AD and is associated with changes in GABAergic neurotransmission^14^. Furthermore, in a mouse model of Down syndrome (DS), where APP is overexpressed, dysregulation of cation Cl^−^ cotransporters expression leads to excitatory GABA_A_R signaling^29^. These DS mice carry an extra copy of mouse chromosome 16 syntenic to the long arm of human chromosome 21^30^ containing the human *APP* gene^31^.

In the present work, the influence of APP on the regulation of NKCC1 and KCC2 expression and effects on GABAergic neurotransmission were studied. We show that NKCC1 expression was not modified in human APP (hAPP) expressing cells, while KCC2 expression was decreased. Using Ca^2+^ imaging and gramicidin perforated patch, we demonstrate that GABA was less hyperpolarizing and less inhibitory in hAPP expressing cells. We conclude that KCC2 expression depends on APP expression levels, involving the juxta-/transmembrane domain of APP and could rely on the KCC2 transcriptional regulator USF1. KCC2 expression was also decreased following hAPP expression *in vivo*.

## Results

### Evolution of KCC2 and NKCC1 expression and GABAergic Ca^2+^ responses during primary cortical culture development

**Figure 1:**
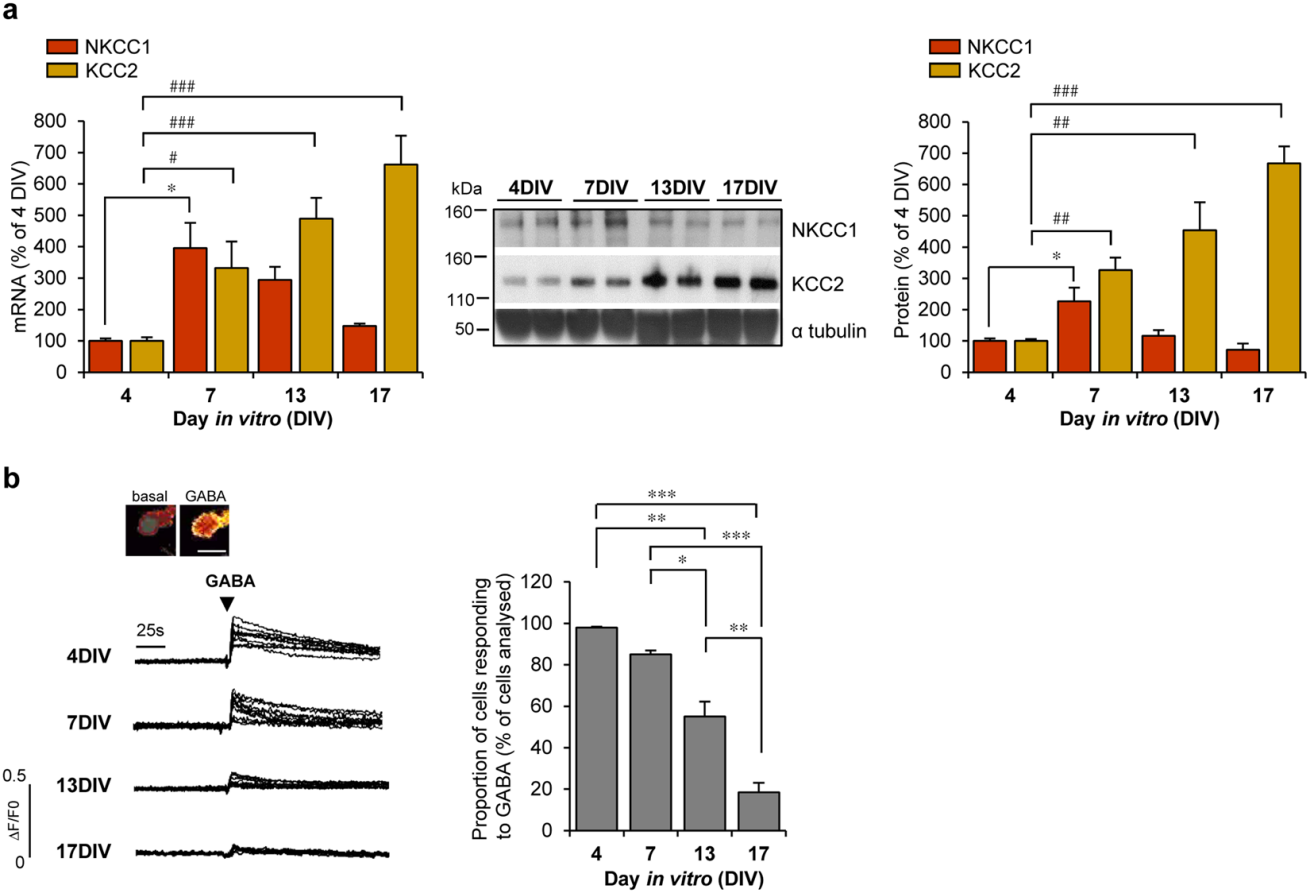
Developmental changes in cations Cl^−^ cotransporters expression and GABA-induced responses in rat cortical cells cultures. (**a**) Left panel: Time course of *Slc12a2* (encoding NKCC1) and *Slc12a5* (encoding KCC2) mRNA levels analysed between 4 and 17 days *in vitro* (DIV) in primary rat cortical cultures by RT-qPCR (n = 7 each analysed in 5 independent experiments). Results were normalized to GAPDH mRNA and expressed as percentage (mean ± s.e.m.) of 4 DIV (*P < 0.05, **P < 0.01, ***P < 0.001, Kruskal-Wallis test followed by Dunn’s multiple-comparison post-test). Middle panel: Representative Western blot of cell lysates from cortical cultures analysed between 4 and 17 DIV. Expression of NKCC1 and KCC2 was monitored using specific antibodies. Blots were further probed with an anti-α tubulin antibody. Right panel: quantification of NKCC1 and KCC2/α tubulin ratios. Results were expressed as percentage (mean ± s.e.m.) of 4 DIV (n = 11 of each analysed in 6 independent experiments, *P < 0.05, **P < 0.01, ***P < 0.001, ANOVA for NKCC1 and Kruskal-Wallis test for KCC2). (**b**) GABA-induced calcium responses in cortical cells during culture development. Top, ratiometric fluorescent dye image in one cell before (basal) and immediately after applying 100 µM GABA. Scale bar, 10 µm. Representative traces of ten cell recordings displaying a [Ca2^+^]_i_ increase in response to GABA. Traces are expressed as ΔF/F0, where ΔF corresponds to a change in F340/F380 ratio and F0 is the basal fluorescence value. Right panel, percentage (mean ± s.e.m.) of cells responding to GABA (n = 215 cells for each time point analysed in 4 independent experiments, *P < 0.05, **P < 0.01, ***P < 0.001, Kruskal-Wallis test).

The transition of GABAergic neurotransmission from depolarizing to hyperpolarizing that takes place not only *in vivo* but also in primary neuronal cultures *in vitro* is associated with a gradual change in NKCC1 and KCC2 expression (for review see ref. 20). Since dynamics and magnitude of these changes depend on the model used, we first decided to fully characterize our experimental model of primary cortical cell cultures. In these cultures, NKCC1 and KCC2 mRNA levels were analysed between 4 and 17 days *in vitro* (DIV). Significant 3- to 4-fold increase in NKCC1 and KCC2 mRNA levels was observed by 7 DIV (Fig. 1a). KCC2 mRNA continued to increase as the culture developed, whereas NKCC1 mRNA gradually decreased after 7 DIV (Fig. 1a). These changes were mirrored by modifications in NKCC1 and KCC2 protein levels (Fig. 1a). As previously reported, the developmental upregulation of KCC2 mRNA and protein levels is essentially due to the KCC2b isoform^32, 33^. The KCC2 and NKCC1 antibodies, as well as the primers used in PCR experiments, recognize both isoforms of KCC2 (KCC2a and KCC2b) and NKCC1 (NKCC1a and NKCC1b).

During the early developmental period, depolarizing GABAergic potentials activate voltage-dependent Ca^2+^ channels (VDCCs) and increase intracellular Ca^2+^ levels ([Ca^2+^]_i_)^34–36^. We monitored GABA-induced Ca^2+^ responses during the maturation of cortical cell cultures by using Fura-2 AM Ca^2+^-sensitive dye (Fig. 1b). Measurements of fluorescence intensity changes showed that cells cultured up to 7 DIV responded to exogenous GABA application (100 μM) with a rapid and reversible increase in [Ca^2+^]_i_. Beyond 7 DIV, the percentage of neurons that responded to GABA decreased with the duration of the culture (Fig. 1b).

Given that by 17 DIV the response to GABA was almost completely gone, depolarization with a high concentration of extracellular KCl (50 mM) still induced robust increases in [Ca^2+^]_i_, suggesting that decreased responsiveness to GABA was due to a decrease in the extent of GABA-induced depolarization (Supplementary Fig. 1a). These observations are consistent with previous findings in cultured neurons^37, 38^. Moreover, GABA responses were blocked by the L-type VDCC antagonist nimodipine (10 μM) (Supplementary Fig. 1b), indicating that the GABA-induced Ca^2+^ increase is mediated by Ca^2+^ influx through L-type Ca^2+^ channels. Furthermore, treatment with the GABA_B_ agonist baclofen (10 µM) did not change [Ca2^+^]_i_, while the GABA_A_ specific agonist isoguvacine (30 µM) increased [Ca2^+^]_i_, indicating that GABA_B_ receptors are not involved in GABA-induced changes in [Ca^2+^]_i_ (Supplementary Fig. 1b). Taken together, the results indicate that maturation related changes in expression of NKCC1 and KCC2 tightly correlate with the evolution of GABA-induced Ca^2+^ responses in our cortical cell model. Furthermore, a shift in GABAergic signaling is apparent in our cultures starting from 7 DIV and GABA induced depolarization is almost completely absent by 17 DIV.

### Human APP expression decreases KCC2 expression and renders GABA signaling less hyperpolarizing and less inhibitory in primary cortical cultures

**Figure 2:**
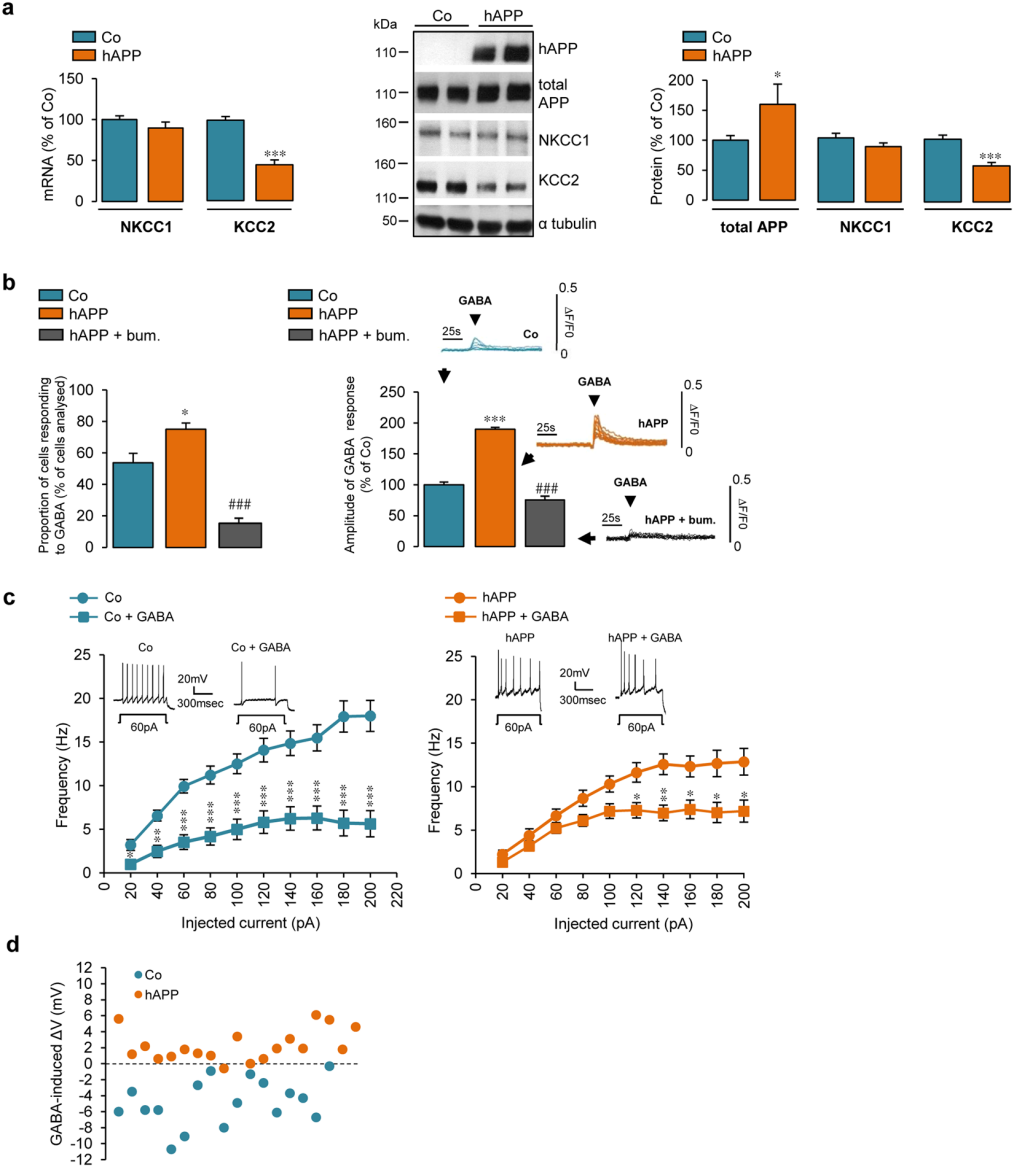
Expression of human APP in cortical cultures decreases KCC2 expression and changes GABA-induced responses. (**a**) Left panel: Comparative RT-qPCR analyses of *Slc12a2* (coding for NKCC1) and *Slc12a5* (coding for KCC2) mRNA levels in primary rat cortical cultures infected or not (control, Co) at 6 DIV with an adenovirus coding for human APP (hAPP). Results were obtained between 13 and 17 DIV (n = 20 for each condition in 9 independent experiments), normalized to GAPDH mRNA and expressed as percentage (mean ± s.e.m.) of Co (***P < 0.001, Student’s *t*-test). Middle panel: Representative Western blot of cell lysates from Co and hAPP cultures. hAPP and total APP, were detected between 13 and 17 DIV with the WO2 and anti-APP C-terminal antibody, respectively. Blots were further probed using anti-NKCC1, -KCC2, and -α tubulin antibodies. Right panel: quantification of total APP, NKCC1 and KCC2/α tubulin ratios expressed as percentage (mean ± s.e.m.) of Co (Co n = 28 and hAPP n = 30 in 16 independent experiments, *P < 0.05, ***P < 0.001, Mann-Whitney test (total APP) and Student’s *t*-test (NKCC1 and KCC2)). (**b**) GABA (100 µM)-induced calcium response in Co and hAPP cultures measured between 13 and 17 DIV incubated with or without 10 µM bumetanide (bum.) for 1 h. Insets: representative traces of ten cell recordings. Traces are expressed as ΔF/F0, where ΔF corresponds to a change in F340/F380 ratio and F0 is the basal fluorescence value. Percentage (mean ± s.e.m.) of cells responding to GABA (left panel) and amplitude of this response (right panel) (Co n = 138, hAPP n = 122, hAPP+ bum. n = 208 cells in 4 independent experiments), ANOVA test followed by Bonferroni’s multiple-comparison post-test (compared to controls: *P < 0.05, ***P < 0.001; to untreated hAPP: ^###^ *P* < 0.001). Frequency and representative traces of action potentials depending on the current injected (**c**) and difference in membrane potential (ΔV) (**d**) in Co and hAPP expressing cortical neurons before or after GABA (100 µM) application measured between 13 and 19 DIV (Co n = 17 and hAPP n = 19 cells in 6 independent experiments, in (**c**) *P < 0.05, **P < 0.01, ***P < 0.001, Student’s *t*-test applied for each current step).

To address whether APP could modulate GABAergic neurotransmission by affecting expression levels of NKCC1 and KCC2, we expressed human APP (hAPP) in cultured rat cortical cells. Full-length neuronal wild-type hAPP expression was achieved by infecting primary cortical cultures with a recombinant adenoviral vector at 6 DIV, i.e. prior to the occurrence of the GABA shift. At 13 DIV, we probed blots with an antibody which discriminates between hAPP and endogenous rat APP by recognizing amino-acids 4–10 of human Aβ, as well as using an antibody that recognizes the 19 carboxyl-terminal amino acids of both human and endogenous APP (total APP) (Fig. 2a). Seven days after infection, expression of hAPP increased the total content of APP by about 1.6 fold (159.6 ± 33%) (Fig. 2a). Moreover transgene expression was homogeneous, did not affect cell density and viability and remained stable over time (Supplementary Fig. 2a–c). Expression of hAPP did not affect NKCC1 expression, but decreased KCC2 mRNA and protein levels by about 50% (44.8 ± 5.3 and 57.4 ± 5.4% of control, respectively) (Fig. 2a). In some cases, oligomeric forms of KCC2 could be detected and decreased to the same extent as the monomeric protein in hAPP expressing cells (46.8 ± 12% of control Supplementary Fig. 3a). Moreover, KCC2 immunofluorescent signal was significantly decreased in hAPP expressing neurons (49.6 ± 4.2% of control, Supplementary Fig. 3b). Infection of cortical cells with an adenovirus encoding human recombinant green fluorescent protein (hrGFP) was not toxic and did not modify expression levels of NKCC1 and KCC2 (Supplementary Fig. 4a and b). In addition, KCC2 and NKCC1 expression remained unchanged in cortical cultures infected with hAPP adenovirus at 13 DIV after the occurrence of the GABA shift (data not shown).

Given that NKCC1 and KCC2 are regulators of neuronal chloride gradients and that these gradients are important for GABAergic transmission polarity, the decrease in KCC2 levels suggested that hAPP expression could have an impact on GABAergic transmission polarity. To test this hypothesis, we monitored GABA-induced depolarization between 13 and 17 DIV by loading control or hAPP expressing cells with Fura-2AM calcium sensitive dye and treating them with GABA (100 µM). As shown in Fig. 2b, hAPP expressing cells displayed increased GABA-induced response as compared to controls. Indeed, the proportion of cells responding to GABA as well as the amplitude of this response were increased in hAPP expressing neurons (75.15 ± 3.9 of total cells analysed and 190.4 ± 2.76% of control, respectively). It is noteworthy that GABA-induced fluorescence was unaffected in hrGFP infected cells (Supplementary Fig. 4c). Furthermore, the increase in [Ca^2+^]_i_ observed in hAPP expressing cells was not due to higher expression of the α3 and α1 GABA_A_R subunits, predominantly expressed in the adult and developing cortex, respectively^39, 40^ (Supplementary Fig. 5a). Since expression of the α1C pore-forming subunit of L-type VDCC (Ca_v_1.2) was previously shown to be modified by APP^41^, we measured Ca_v_1.2 expression as well as another pore forming subunit of L-type channels, α1D (Ca_v_1.3) in hAPP expressing primary cortical cells. Ca_v_1.2 and Ca_v_1.3 expression remained unchanged in hAPP expressing cells (Supplementary Fig. 5b).

In order to confirm that the GABA-induced rise in [Ca^2+^]_i_ observed in hAPP expressing neurons was sensitive to changes in chloride concentration, we incubated primary cortical neurons with the NKCC1 inhibitor bumetanide^42^. It was assumed that by decreasing [Cl^−^]_i_, bumetanide would render GABA more hyperpolarizing which in turn would result in a reduced GABA-induced Ca^2+^ response. As expected, in hAPP expressing neurons treated with 10 µM bumetanide^42^, the proportion of cells responding to GABA as well as the amplitude of the GABA response were decreased compared to untreated hAPP expressing cells (19.63 ± 5.7% of total cells analysed and 50.3 ± 4.6% of untreated hAPP) (Fig. 2b), indicating that changes in [Cl^−^]_i_ may contribute to GABA-induced increase in [Ca^2+^]_i_ observed in hAPP expressing neurons. Taken together the results suggest that GABA is more depolarizing in hAPP expressing neurons, which could be due to a modification in [Cl^−^]_i_ levels resulting from a decrease in KCC2 expression in these cells.

Given the increased depolarization evoked by GABA in hAPP expressing neurons observed by calcium imaging, gramicidin perforated patch recordings in hAPP expressing neurons were performed in order to evaluate the effect of GABA (100 µM) on evoked action potential (AP) frequency and resting membrane potential between 13 DIV and 19 DIV. As expected, addition of GABA reduced AP frequency in control neurons by about 65% (Fig. 2c). On the other hand in hAPP expressing neurons, AP frequency was reduced only by about 35% indicating that GABA was less inhibitory in these cells (Fig. 2c). Furthermore, while application of GABA elicited a significant hyperpolarization in most control cells analysed (ΔVm −4.83 mV ± −0.55), hAPP expressing neurons either slightly depolarized or did not display a change in membrane potential in response to GABA (ΔVm 2.25 mV ± 0.36) (Fig. 2d and Supplementary Fig. 6a). The effect of GABA measured by patch clamp in neurons infected with hrGFP was not significantly different compared to controls (Supplementary Fig. 6b and c). These results support the idea that GABA is more depolarizing in hAPP expressing neurons compared to controls. However, the more depolarizing effect of GABA in hAPP expressing cells is not sufficient to elicit excitation. Indeed, GABA remained inhibitory in hAPP expressing neurons, but to a lesser extent than in controls.

### APP dependent KCC2 downregulation in primary cortical cultures involves the juxta-/transmembrane domain of APP and could rely on USF1

**Figure 3:**
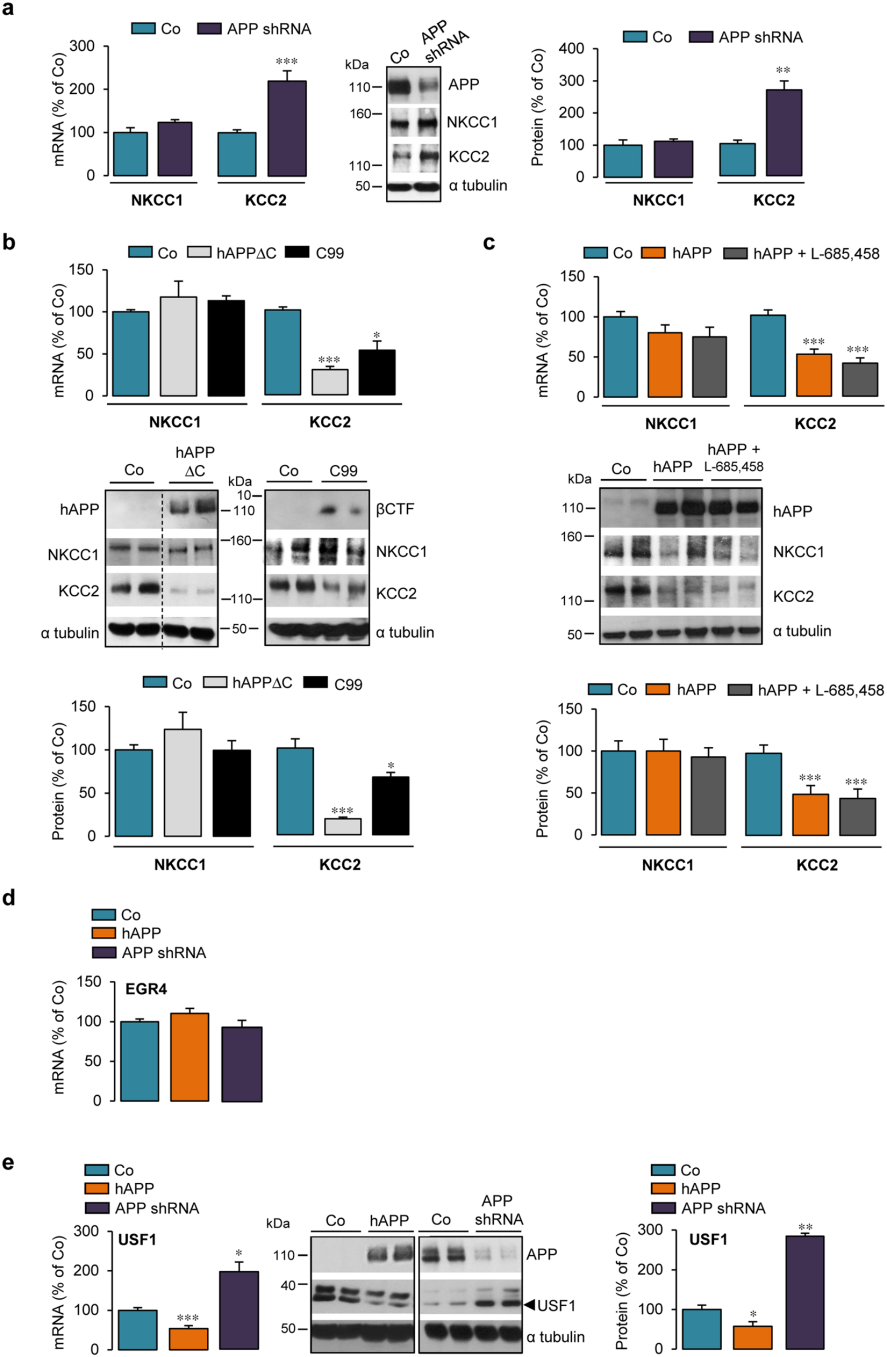
APP dependent KCC2 downregulation in primary cortical cultures involves juxta-/transmembrane domain of APP and could rely on USF1. (**a**–**e**) Comparative RT-qPCR analyses of *Slc12a2* (encoding NKCC1) and *Slc12a5* (encoding KCC2) (**a**–**c**) or EGR4 and USF1 (**d**,**e**) mRNA levels in primary rat cortical cultures infected or not (control, Co) at 6 DIV with shRNA APP lentiviruses (**a**,**d**,**e**) or adenoviruses encoding human (hAPP) truncated in its carboxyl- or amino terminal-domain (b: hAPPΔC, C99, c: hAPP). In (**c**) hAPP expressing cells were treated with 1 µM L-685,458 γ-secretase inhibitor for 24 h. Analysis was performed between 13 and 17 DIV. Results were normalized to GAPDH mRNA. (**a**–**c**,**e**) Representative Western blots of cell lysates from cortical cultures infected or not (control, Co) at 6 DIV with shRNA APP lentiviruses (**a**,**e**), hAPPΔC, C99 or hAPP adenoviral constructs (in cropped Western blots **b** and **c**). Between 13 DIV and 17 DIV, expression of hAPP and βCTF was monitored with the specific WO2 antibody and expression of endogenous APP was assessed using the anti-APP C-terminal antibody. Blots were further probed using anti-NKCC1, -KCC2, USF1 and -α tubulin antibodies. NKCC1, KCC2 and USF1 signals were normalized to α tubulin, quantified and expressed as percentage (mean ± s.e.m.) of Co: *P < 0.05, **P < 0.01, ***P < 0.001; (**a**) Student’s *t*-test for NKCC1 and Mann-Whitney test for KCC2; (**b**) Kruskal-Wallis test; (**c**,**e**): ANOVA (except for USF1 mRNA in (**e**) Kruskal-Wallis test. (**a**) Co n = 8 and APPshRNA n = 14 (mRNA) and Co n = 5 and APPshRNA n = 7 (protein) analysed in 5 and 3 independent experiments, respectively; (**b**) Co n = 11, APPΔC and C99 n = 7 of each in 5 independent experiments; (**c**) hAPP n = 7 and hAPP+ L-685,458 n = 8, each in 3 independent experiments. In d and e, hAPP and Co n = 11 in 5 independent experiments; APPshRNA n = 6 (mRNA) and n = 11 (protein) in 3 independent experiments.

We next investigated whether KCC2 downregulation was related to APP expression level. NKCC1 and KCC2 expression was first measured in cultured cortical cells from wild type (*App* ^+/+^) and APP deficient (*App* ^−/−^) mice. At 13 DIV, the absence of APP and/or its cleavage products did not modify NKCC1 and KCC2 mRNA and protein levels (Supplementary Fig. 7a). Nevertheless, tendency towards increased KCC2 expression was observed. Given that APP belongs to a multi-gene family of paralogs including amyloid precursor-like proteins (APLPs) 1 and 2^43^, APLP1 and APLP2 could functionally compensate for the loss of APP^44, 45^. Indeed, we observed a non-significant increase in APLP1 expression in *App* ^−/−^ compared to *App* ^+/+^ cells, suggesting partial compensation of APP deletion by APLP1 (Supplementary Fig. 7b). To bypass compensatory mechanisms that might have masked APP function, APP expression was reduced in cortical cells using an shRNA construct designed to target endogenous APP (APP shRNA). The knock-down of endogenous rat APP was achieved by infecting cells on 6 DIV with recombinant lentiviruses encoding APP shRNA. At 13 DIV, blots were probed with an antibody that recognizes the 19 carboxyl-terminal amino acids of endogenous APP (Supplementary Fig. 8a). One week after APPshRNA lentiviral infection, a 68.8 ± 8.6% decrease in endogenous APP expression was observed, while APLP1 and APLP2 protein levels remained unchanged (Supplementary Fig. 8a). Using this knock-down approach, NKCC1 expression was not modified after APPshRNA lentiviral infection (Fig. 3a). By contrast, a significant increase in KCC2 mRNA and protein levels (219.3 ± 24.2 and 268.2 ± 28.1% of control, respectively), was observed (Fig. 3a). Moreover, infection of cortical cells with a recombinant lentivirus encoding neomycin resistance (neo) did not change KCC2 protein levels (Supplementary Fig. 8b). The results thus indicate that KCC2 expression depends on APP expression.

We next investigated whether cleavage products of APP could be involved in the regulation of KCC2 expression. APP is a type-I transmembrane protein with a large extracellular domain and a short intracellular domain (Supplementary Fig. 9), both contributing to APP function (for review, see ref. 5). We first tested whether the APP intracellular domain (AICD), a transcriptional regulator^46^, could be involved in the downregulation of KCC2. Primary cortical cell cultures were infected with a recombinant adenovirus encoding a hAPP variant containing the APP extracellular and transmembrane domains, but lacking its intracellular domain (APPΔC) (Fig. 3b). Expression of APPΔC greatly decreased KCC2 mRNA and protein levels (Fig. 3b) (29.4 ± 3.8 and 18.3 ± 1.8% of control, respectively). Moreover, KCC2 expression was unaffected in cells infected by a control adenovirus encoding β-galactosidase (β-gal, Supplementary Fig. 10). Taken together, the results ruled out involvement of AICD in the hAPP dependent KCC2 decrease.

Using a similar strategy, the question of whether the extracellular domain of APP could play a role in regulating KCC2 expression was investigated. An adenoviral vector encoding the transmembrane and intracellular domains of hAPP i.e. the naturally occurring APP C99 fragment, which begins at the β-secretase cleavage site (CTFβ) (Supplementary Fig. 9) was used. Expression of C99 decreased KCC2 mRNA and protein levels to a similar extent as observed with full-length hAPP (52.3 ± 10.8 and 66.5 ± 5.3% of control, respectively) (Fig. 3b), indicating that the hAPP extracellular domain does not play a role in the hAPP-dependent regulation of KCC2 expression.

γ-secretase processing of APP is physiologically important since it leads not only to AICD production but also to that of Aβ, which plays an important role in the pathophysiology of AD and has been assigned many functions^6^. Involvement of γ-secretase processing in hAPP dependent KCC2 downregulation was studied using a highly potent and selective γ-secretase inhibitor, L-685,458 (1 µM)^47^. Inhibition of γ-secretase activity for 24 h in hAPP expressing cells significantly decreased extracellular human Aβ 1-40 levels (Supplementary Fig. 11a) and induced APP carboxyl-terminal fragments (CTFs) accumulation (Supplementary Fig. 11b), but did not affect NKCC1 and KCC2 mRNA and protein levels (Fig. 3c). Together, the results indicate that the hAPP-mediated decrease in KCC2 is AICD and γ-secretase activity independent and does not involve the hAPP extracellular domain.

The KCC2b promoter has 10 putative transcription factor binding sites^48^. Amongst these, the only two transcription factors clearly described as potent regulators of *Slc12a5* gene encoding KCC2 are early growth response 4 (EGR4) and upstream stimulating factor 1 (USF1)^48, 49^. Given that hAPP is able to decrease KCC2 mRNA levels, expression of KCC2 transcriptional regulators EGR4 and USF1 was measured in hAPP expressing cells. Although EGR4 can modulate KCC2 expression in developing neurons^48^, EGR4 mRNA level was unchanged in hAPP expressing cells and in cultures infected with APPshRNA lentiviral vector (Fig. 3d). By contrast, a 50% decrease in USF1 mRNA was seen in hAPP expressing cells (53.5 ± 7.7% of control, Fig. 3e). Unfortunately, commercially available EGR4 antibodies did not detect the EGR4 protein in our cortical cell extracts. On the other hand, USF1 protein levels were decreased in hAPP expressing cells (57.8 ± 11.5% of control) (Fig. 3e). Moreover, mRNA and protein levels of USF1 were increased in cultures infected with an APPshRNA lentiviral vector (201 ± 25.2 and 282 ± 7.7% of control, respectively) (Fig. 3e). Taken together, the results suggest that APP can modulate USF1 and KCC2 expression in a similar manner providing a hint to a putative mechanism of APP dependent KCC2 expression regulation.

### KCC2 expression is decreased in AAV hAPP transduced mouse brains

**Figure 4:**
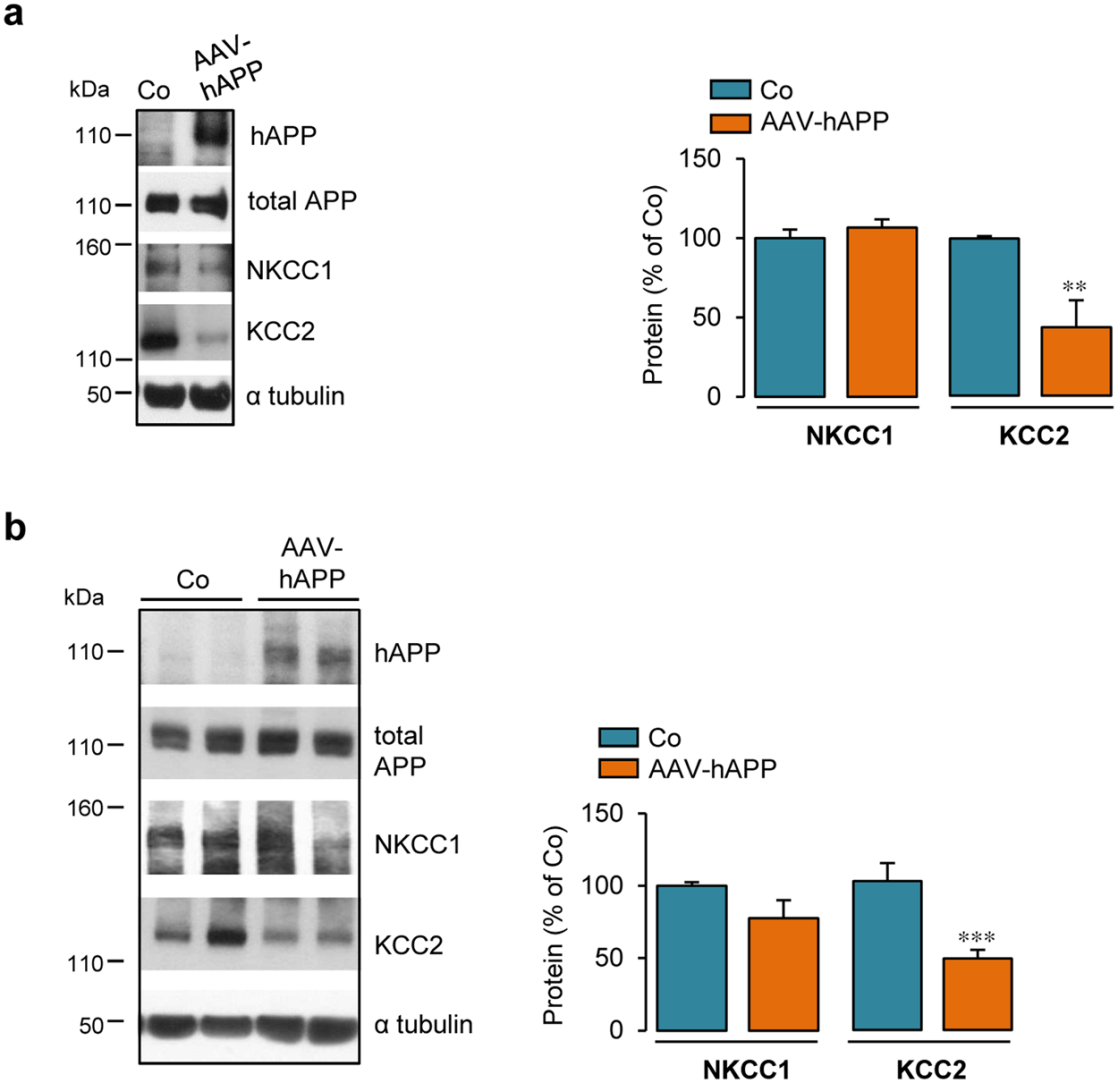
NKCC1 and KCC2 expression in AAV-hAPP transduced cortical cells and mouse brains. (**a**) Representative Western blot of cell lysates from primary rat cortical cultures infected or not (control, Co) at 6 DIV with serotype 2 associated-adenoviruses encoding wild-type human APP (AAV-hAPP). At 13 DIV, expression of hAPP was monitored with the specific WO2 antibody and expression of both hAPP and endogenous APP (total APP) was analysed using the anti-APP C-terminal antibody. Blots were further probed using anti-NKCC1, -KCC2 and -α tubulin antibodies. Right panels: quantification of NKCC1 and KCC2/α tubulin ratios. Results were expressed as percentage (mean ± s.e.m.) of Co (n = 3 of each analysed in 3 independent experiments, **P < 0.01, Student’s *t*-test except for KCC2 in (**a**): Mann-Whitney test. (**b**) Representative Western blot of brain homogenates of mice intraventricularly injected at P1-P2 with AAV-hAPP. 30 days post injection, expression of hAPP was monitored with the specific WO2 antibody and expression of both hAPP and endogenous APP (total APP) with the anti-APP C-terminal antibody. Blots were further probed using anti-NKCC1, -KCC2, and -α tubulin antibodies. NKCC1 and KCC2/α tubulin ratios were quantified (right panel). Results were expressed as percentage (mean ± s.e.m.) of Co (***P < 0.001; Mann-Whitney test). Co n = 7 mice and AAV-hAPP n = 5 mice.

Since adenoviral hAPP transduction decreased KCC2 expression *in vitro*, we wondered whether hAPP transduction could affect KCC2 and NKCC1 levels *in vivo*. Therefore, serotype 2 associated-adenoviruses encoding full-length neuronal wild-type human APP and green fluorescent protein were produced (AAV-hAPP and AAV-GFP, respectively). AAV constructs were first tested *in vitro* by infecting cultured cortical cells at 6 DIV. At 13 DIV, hAPP and GFP could be detected in AAV-hAPP and AAV-GFP infected cells by Western blotting, respectively (Fig. 4a and Supplementary Fig. 12a, respectively). Moreover, we show that hAPP expression, while not affecting NKCC1 expression, decreased KCC2 protein levels by more than 50% (43.6 ± 17.1% of control) (Fig. 4a). Furthermore, infection of cortical cells with AAV-GFP did not modify expression levels of neither NKCC1 nor KCC2 (Supplementary Fig. 12a).

A shift in polarity of GABA_A_ responses was shown to occur during the first two postnatal weeks in rodent hippocampus^50^. Moreover, unlike in the human neocortex^51, 52^, KCC2 is robustly upregulated perinatally in rodents and continues to increase during the first postnatal month^20, 53^. Based on these observations and in order to mimic the timing of adenoviral transductions made *in vitro* (6 DIV), mice pups were intraventricularly injected with an AAV-hAPP or -GFP construct on postnatal day 1 or 2 (P1-P2), i.e. prior to the occurrence of the GABA_A_ transmission polarity shift^50^. Brains of injected mice pups were recovered at P30, the time point when cortical synaptic maturation had been completed (for a review see ref. 54). By western-blotting, hAPP expression in the posterior part of cerebral hemispheres of AAV-hAPP injected mice was examined (Fig. 4b). In these samples, KCC2 protein expression decreased while NKCC1 expression remained unchanged (Fig. 4b). Furthermore, protein levels of NKCC1 and KCC2 were not affected by AAV-GFP injection (Supplementary Fig. 12b). These results indicate that hAPP was able to decrease KCC2 expression both *in vitro* and *in vivo*.

## Discussion

In this study, the influence of APP on regulation of NKCC1 and KCC2 expression with an emphasis on GABAergic neurotransmission was investigated. It was shown that hAPP was able to decrease KCC2 expression both *in vitro* and *in vivo*. Furthermore, the decrease in KCC2 expression observed *in vitro* was associated with a less inhibitory and less hyperpolarizing GABA signaling. In addition, our study unveiled an involvement of the juxta-/transmembrane domain of APP in this regulation.

Several lines of evidence coming from mice overexpressing or lacking APP indicate that APP could play a role in GABAergic neurotransmission^10–15, 55^. Our study investigated the link between APP and indirect GABAergic neurotransmission actors - cation-chloride cotransporters NKCC1 and KCC2. Our results show that expression of human APP was able to decrease KCC2 without modifying NKCC1. Deidda *et al*. have previously shown that in a mouse model of Down’s syndrome NKCC1 expression is increased and leads to excitatory GABA_A_R signaling^29^. These mice have an extra copy of mouse chromosome 16 which is syntenic to the long arm of human chromosome 21^56, 57^ and therefore carry 3 copies of APP gene^58, 59^. In this study, no significant KCC2 expression change was observed. Even though APP is overexpressed in these mice, the genomic region triplicated in this model codes for a whole range of genes besides APP that could be able to influence cation-chloride cotransporters expression. In a recent study by Chen and al., a decrease in KCC2 protein levels was observed in *APP* ^−/−^ mice^55^. The authors have shown that APP and KCC2 can interact and that APP functions to limit tyrosine-phosphorylation and ubiquitination and thus subsequent degradation of KCC2. While in our study we have analyzed the impact of APP on KCC2 expression around the timing of the GABA shift occurrence, we were also interested to see whether APP could influence KCC2 levels in adult mice. Therefore, we have analyzed expression levels of KCC2 in the cortices of *App* ^−/−^ mice at several ages. At the age of 8 months, we observed indeed a decrease in KCC2 protein in *App* ^−/−^ mice (data not shown), confirming the results of Chen *et al*.^55^. In contrast, at the age of 3 months, the absence of APP induced a slight increase in KCC2 expression, in particular at the mRNA level (data not shown), indicating that APP can regulate KCC2 protein level by different mechanisms, depending on the age studied.

As illustrated by evoked action potential measurements in hAPP expressing primary cortical cultures, GABA dependent inhibition as well as GABA-induced hyperpolarization were decreased. However, we recorded spiking activity using cell-attached mode from the cell bodies of hAPP expressing neurons and therefore addressed changes only in their global excitability. Given this we cannot exclude that specific cell compartments like certain neuronal processes may respond to GABA differently as KCC2 expression pattern could be modified by hAPP^60, 61^. Furthermore, we can’t rule out that reduced KCC2 expression could be compensated by a change in its post-translational regulation (for review^62^). Although we measured no change in expression levels of GABA_A_R subunits α3 and α1, the function of theses receptors could be modified by hAPP.

Numerous physiological functions have been assigned to cleavage products of APP. Therefore, we studied which part of hAPP could be important for reduced KCC2 expression. Constructs lacking the APP intracellular domain (APPΔC) and extracellular domain (C99) were expressed in primary cortical cells and both constructs decreased KCC2 expression. However, APPΔC decreased KCC2 expression to a greater extent than full-length hAPP. This difference could be due to a trafficking disparity between APPΔC and full-length APP^63^. For example, the APP C-terminal domain contains the YENPTY internalization motif which is required for APP endocytosis necessary for APP processing^5^. Experiments using APPΔC and C99 supported the idea that the juxta-/transmembrane domain of APP containing Aβ sequence would be important for APP dependent KCC2 regulation. This domain not only contains Aβ sequence, but also contributes to APP dimerization (reviewed in ref. 64). Furthermore, even though the γ-secretase inhibitor L-685,458 did not affect KCC2 expression, we cannot exclude that Aβ could modify KCC2 expression. Indeed, after L-685,458 treatment, residual Aβ was still detectable by ECLIA. It is noteworthy that decreased KCC2 expression has been observed in the hippocampus AD11 mice which express an anti-NGF antibody and develop AD-like hallmarks including amyloid deposits^65^. In addition, mice overexpressing p60TRP, a G-protein coupled receptor known to promote non-amyloidogenic processing of APP, bare an increase in KCC2 expression^66^. Given these observations, it would be interesting to investigate whether these KCC2 expression changes could be linked to a dysregulation in APP processing.

Given that hAPP altered KCC2 mRNA levels, we investigated whether expression of potent transcriptional regulators of KCC2 (EGR4 and USF1) could be modified by APP^48, 49^. EGR4 mRNA levels did not change in hAPP expressing neurons, whereas a correlation between USF1 and KCC2 expression was observed in these cells. Nevertheless, as EGR4 protein was undetectable, we cannot assess whether its expression level would be unchanged in hAPP expressing cells. Moreover, even if the correlation we observed could implicate USF1 in APP dependent KCC2 regulation, there are nine other transcription factor binding sites located in the KCC2 promoter^48^.

Although APP is not overexpressed in sporadic AD, APP is an important player in this pathology where its metabolism and/or function are highly impaired. Therefore, we wondered whether KCC2 and NKCC1 expression could be modified in sporadic AD. We extended our analysis by examining KCC2 and NKCC1 expression in frontal cortex brain tissues from human control subjects and patients suffering from dementia clinically diagnosed with late onset AD (Supplementary Table 1). While NKCC1 expression remained unchanged, we detected a significant decrease in KCC2 expression in the brains of these patients (Supplementary Fig. 13). A potential involvement of Aβ in APP dependent KCC2 regulation cannot be excluded and one could speculate that increased levels of Aβ and/or an alteration in the physiological function of APP could influence KCC2 expression in AD. Nevertheless, AD is a complex disease involving numerous factors that could contribute to the changes we observed. Indeed, in AD there is loss of cholinergic neurons and it is known that KCC2 can be regulated by cholinergic neurotransmission^67^. Furthermore, excitability problems in AD have been noticed (i.e. increased risk for epilepsy) (reviewed in ref. 68) and KCC2 expression can be regulated by neuronal activity^62^. In addition, another important actor in AD, the microtubule associated protein tau, was shown to influence GABAergic transmission and might therefore influence levels of KCC2^69^. It is noteworthy that KCC2 and NKCC1 expression or functional changes have been observed in many other pathologies including epilepsy^62^, autism^70^, Fragile X syndrome^71^, traumatic brain injury^72^, Down syndrome^29^ where an APP/Aβ dysregulation has been described^7, 31, 73^.

Our study adds yet another dimension to the previous work as it highlights the involvement of APP in the regulation of KCC2 expression, which could influence the polarity of GABAergic neurotransmission. Moreover, it should be mentioned that such regulation could also be extended to the excitatory system. Indeed, KCC2 plays an important structural role in dendrites independent of its Cl^−^ transport function making it a synchronizing factor in the development of both inhibitory and excitatory neurotransmission^74^. KCC2 is involved in the functioning and development of synapses and could help to stabilize them. Given that APP is known to modulate neurite outgrowth and synapse development^75, 76^, one might speculate that APP-dependent KCC2 regulation plays a role in these mechanisms. Furthermore, our study introduces a potential developmental aspect for APP dependent KCC2 regulation. Indeed, decreased KCC2 expression was observed only when hAPP adenoviral infection was performed at 6 DIV, prior to the appearance of the GABA shift, suggesting that hAPP expression might block the developmental upregulation of KCC2 in primary cortical cultures. Therefore, it would be interesting to study the involvement of APP in inhibitory and excitatory synapse development with a focus on KCC2.

In conclusion, our results point to a new important role of APP in the regulation of KCC2 expression, which could influence GABAergic neurotransmission. This information advances our understanding of the physiological function of APP and sheds light on new mechanisms that could contribute to AD pathology.

## Methods

### Animals

All animal procedures used in the study were carried out in accordance with institutional and European guidelines and experimental protocols were approved by the Animal Ethics Committee from the Université catholique de Louvain (UCL, Brussels, Belgium). *App* ^−/−^ transgenic mice^77^ were kept on C57Bl6/J genetic background and obtained from Jackson Laboratories (strain name: B6.129S7-Apptm1Dbo/J). Animals were housed on a 12 hour light/dark cycle in standard animal care facilities. Both pregnant Wistar rats used for embryonic cortical cell cultures and mice pups of either sex used for intraventricular injections (C57Bl6/J genetic background) were obtained from the Université catholique de Louvain (UCL, Brussels, Belgium) animal facility.

### Culture reagents and antibodies

Unless specified, reagents for cell culture, western blotting and calcium imaging were purchased from Thermo Fisher Scientific (Waltham, MA, USA). Antibodies were from the sources indicated: mouse monoclonal WO2 anti-hAPP, rabbit polyclonal anti-KCC2 and rabbit polyclonal anti-APLP1 and APLP2 (EMD Millipore, Billerica, MA, USA, catalog nos. MABN10, 07–432 and Calbiochem catalog nos. 171615 and 171616, respectively); mouse monoclonal anti-NKCC1 (Developmental Studies Hybridoma Bank, Iowa city, IA, USA, catalog no. T4); rabbit polyclonal anti-APP C-terminus and mouse monoclonal α tubulin, (Sigma-Aldrich, St-Louis, MO, USA, catalog nos. A8717 and T6074, respectively); rabbit polyclonal anti-hrGFP (Agilent Technologies, Santa Clara, CA, USA, cat no. 240141) and anti-GFP (Sigma-Aldrich, St-Louis, MO, USA, catalog no. AB10145); rabbit anti-GABA_A_R subunits α1 and α3 (Alomone Labs, Jerusalem, Israel, catalog nos AGA-001 and AGA-003, respectively); mouse monoclonal anti-CaV1.3 and anti-CACNA1C (Abcam, Cambridge, UK, cat nos; ab85491, and ab84814, respectively) and rabbit anti-USF1 (C-20) (Bio-Connect life sciences, Huissen, The Netherlands, catalog no. sc-229).

### Cell culture

17–18 day-old embryos of either sex were taken from Wistar rats or wild type and *APP* ^−/−^ mice euthanized with CO_2_. Cortices were isolated and gently disrupted by passing the tissue through a Pasteur pipette with a fire-polished tip. The cells were plated in culture dishes (4 × 10^5^ cells/cm^2^) pre-treated with 10 µg/ml poly-L-lysine in phosphate buffered saline (PBS) and cultured for 6 days *in vitro* in Neurobasal medium supplemented with 2% (v/v) B-27 medium and 0.5 mM L-glutamine prior to infection with recombinant viruses. The cultures were maintained at 37 °C under a 5% CO_2_ atmosphere.

### Preparation of recombinant viruses and infection of cell culture

#### Adenoviruses

Recombinant adenoviruses encoding wild-type human APP695 (AdhAPP), β-cleaved C-terminal fragment of hAPP fused to the APP signal peptide (AdC99) and human recombinant Green Fluorescent Protein-2 (AdhrGFP) were prepared as described previously using the AdEasy^TM^ XL Adenoviral Vector System (Stratagene, La Jolla, CA, USA, catalog no. 240010)^78^. Briefly, either the pShuttle-CMV-internal ribosomal entry site (IRES)-hrGFP-2 vector (for AdGFP) or the same vector containing the cDNA encoding either human APP695 or its C-terminal region (residues 597 to residue 695 for AdC99) was cloned into the pAdEasy-1 vector. Adenoviruses were purified according to the manufacturer’s instructions.

The construction and purification of recombinant adenoviruses encoding β-galactosidase (Adβgal) and human APP695 truncated at its C-terminal region from residues 652 to 695 (AdhAPPΔC) were made as described previously^78^. The AdhAPPΔC construct contains a stop codon after the three lysine residues that follow the APP transmembrane domain. Cells infected with AdhrGFP and Adβgal were used as control. After 6 days *in vitro* (6 DIV), the cells were infected at a multiplicity of infection (MOI) of 10 in a minimal volume of culture medium for 4 h. Infection medium was replaced by fresh culture medium every two days up to analysis (between 13 DIV and 19 DIV).

#### Lentiviruses

Two different pLKO.1 vectors encoding shRNA targeting APP mRNA were obtained from Sigma-Aldrich, St-Louis, MO, USA, (catalog nos. TRCN0000054874 Clone ID: NM_007471.2-2185s1c1 and TRCN0000054876 Clone ID: NM_007471.2-1583s1c1, respectively) and used for construction of recombinant lentiviruses, as previously described^79^. Briefly, lentiviruses were produced by calcium phosphate cotransfection of the target plasmid together with lentiviral packaging plasmids (gag-pol, rev, and VsVg plasmids, a kind gift from Pr. T. Michiels (de Duve Institute, Brussels, Belgium)) into HEK293T/17 cells (ATCC^®^, Manassas, VA, USA, catalog no. CRL-11268^TM^). Supernatants containing the lentiviruses were harvested 48 h after transfection. Lentiviruses were concentrated and purified with the Lenti-X ™ Concentrator kit (Clontech Laboratories, Mountain View, CA, USA, catalog no. PT4421-2) according to the manufacturer’s instructions. Aliquots (30 µl) of concentrated lentiviral solution were added to cell cultures on 6 DIV. A recombinant lentivirus encoding the neomycin resistance gene (pTM898neo, a kind gift from Pr. T. Michiels (de Duve Institute, Brussels, Belgium)) was used as a negative control. Cells were analysed on 13 DIV.

#### Adeno Associated Viruses (AAV)

pAAV-MCS Vector of AAV-DJ/8 Helper Free Expression System was used in order to clone hAPP (Cell Biolabs Inc., San Diego, CA, USA catalog no. VPK-410-DJ-8). This pAAV-hAPP vector contains inverted terminal repeats of an AAV2 sequence bordering a CMV promoter driving expression of hAPP. A control AAV vector pAAV-CMV-GFP was obtained from Connie Cepko (Addgene, Cambridge, MA, USA, catalog no. #67634). pHelper and pRC2-mi342 plasmids used for AAV cross-packaging were obtained from AAVpro Helper Free System (AAV2) (Takara Bio Europe/Clontech, Saint-Germain-en-Laye, France, catalog no. #6230). AAV2 particles were generated based on the AAVpro Helper Free System protocol. Briefly, HEK 293 T/17 cells (ATCC^®^, Manassas, VA, USA, catalog no. CRL-11268^TM^) were transfected with 45 µg total of pAAV-hAPP or pAAV-CMV-GFP, pHelper and pRC2-mi342 plasmids (ratio 1:1:1) using the Mirus TransIT 293 (Mirus Bio, Madison, WI, USA, catalog no. MIR 2700) transfection reagent (3 µl/1 µg of DNA). After 72 hours of transfection, cell pellets were recovered and AAV2 viral particles were extracted using the AAVpro® Extraction Solution (Takara Bio Europe/Clontech, Saint-Germain-en-Laye, France, catalog no. #6235). AAV2 particles dedicated to *in vivo* usage were purified using the ViraBind™ AAV Purification Mega Kit (Cell Biolabs, inc. San Diego, CA, USA, catalog no. #VPK-141). The genomic titer of the virus was measured using the AAVpro® Titration Kit (for Real Time PCR) Ver.2 (Takara Bio Europe/Clontech, Saint-Germain-en-Laye, France, catalog no. #6233). Cultures were transduced on 6 DIV using AAV2-hAPP or AAV2-GFP at an MOI of 3000. A maximum of 100 µl microliters of crude viral extract or purified virus was added to each well of a 12 well plate containing approximately 900,000 cells in 1 ml.

For AAV2 - hAPP and - GFP transduction in neonatal mouse brain, one or two days after birth (P1-P2), C57Bl6/J neonates were cryoanesthetized and injected using a 10 µl Hamilton glass syringe (Filter Service, Eupen, Belgium, catalog no. HA 7635-01) at an angle of 30 °C to a depth of 1.5 mm. Purified concentrated viral vector (2 μl) was slowly injected into each cerebral lateral ventricle. A total of 4.5 × 10^8^ genomic equivalents (4 μl) was injected into each mouse brain. After injection, pups were allowed to completely recover on a warming blanket and then returned to the home cage for later analysis.

#### Treatments

Cells were treated for 1 h with bumetanide 10 µM (Sigma-Aldrich, St-Louis, MO, USA, catalog no. B3023) prior to Ca^2+^ imaging. On 12 DIV and for 24 h, cells were treated with (5 S)-(t-Butoxycarbonylamino)-6-phenyl-(4 R)hydroxy-(2 R)benzylhexanoyl)-L-leu-L-phe amide (L-685,458, 1 µM), a γ-secretase inhibitor (Sigma-Aldrich, St-Louis, MO, USA, catalog no. L1790).

#### Western blotting

Cells in culture were washed, scraped off into PBS and centrifuged (10,000 rpm × 2 min). The cell pellets were sonicated in lysis buffer (125 mM Tris (pH 6.8), 20% glycerol, and 4% sodium dodecyl sulphate) supplemented with complete Protease Inhibitor Cocktail (Roche, Bâle, Switzerland). For brain protein extraction, samples were homogenized in RIPA buffer (1% (w/v) NP40, 0.5% (w/v) deoxycholic acid, 0.1% (w/v) SDS, 150 mM NaCl, 1 mM EDTA, 50 mM Tris, pH 7.4) containing 1 mM PMSF, 10 mM NaF, 2 mM sodium orthovanadate and 1% (v/v) protease and phosphatase inhibitor cocktail (Roche, Basel, Switzerland). The samples were clarified by centrifugation at 20 000 g and protein concentrations were determined using a Bicinchoninic Acid Assay (BCA) kit. Samples were heated for 10 min at 70 °C in loading buffer (lysis buffer containing 10% (w/v) 2-Mercaptoethanol and 0.004% (w/v) bromophenol blue).

Cell and brain lysates (20 µg and 40 µg of proteins, respectively) were subjected to SDS-PAGE using 4–12% Nupage^TM^ bis-Tris gels for Western blotting. Nitrocellulose membranes were incubated overnight at 4 °C with the following primary antibodies: human APP-specific WO2 (1:2 000); anti-APP C-terminal (1:2 000); anti-KCC2 (1:1 000); anti-hrGFP and GFP (1:2 000), anti-NKCC1 (1:100); anti-GABA_A_R subunits α1 and α3 (1:2 000 and 1:1 1000; respectively); anti-CaV1.3 (1:1 000), anti-CACNA1C (1:1 000), anti-α tubulin (1:4 000), anti-APLP1 and APLP2 (1:4 000 and 1:2 000, respectively) and anti-USF1 (1:500). Blots were incubated with horseradish peroxidase-conjugated secondary antibodies, revealed by ECL (Amersham Pharmacia), and quantified using the Quantity One^TM^ software (Bio-Rad Laboratories, Hercules, CA, USA) with α-tubulin as a loading control.

#### RNA extraction and real time PCR

Total RNA was isolated by TriPure Isolation Reagent according to the manufacturer’s protocol. RNA samples were resuspended in DEPC-treated water. Reverse transcription was carried out with the iScript cDNA synthesis Kit (Bio-Rad Laboratories, Hercules, CA, USA, catalog no. 1708891) using 1 µg of total RNA in a total reaction volume of 20 μL. Controls were performed without reverse transcriptase to rule out amplification of contaminant genomic DNA. Real-time PCR was performed for the amplification of NKCC1, KCC2, EGR4, USF1 and glyceraldehyde phosphate dehydrogenase (GAPDH) cDNAs. Primers were purchased from Sigma-Aldrich (St-Louis, MO); **F**, Forward primer; **R**, Reverse primer; **E**, primer efficiency:

NKCC1 rat

**E**-107.2%, **F**-5′TTCGTGAGAGGAGGAGGAG3′, **R**-5′GATGCCCAGAAGAACCAC3′

KCC2 rat and mouse

**E**-102.0%, **F**-5′GGTGGAAGTCGTGGAGATG3′, **R**-5′CGAGTGTTGGCTGGATTC3′

USF1 rat

**E**-95.7%, **F-**5′TGATGATGCTGTTGACGC3′, **R-**5′TGGCTCCCTCCCTGTAAC3′

EGR4 rat

**E**-108.2%, **F**-5′CGACGAGAAGAAGCGACA3′, **R**-5′CCAGCGAGTAGAAGCCCA3′

GAPDH rat/mouse

**E**-106.7%, **F**-5′CCCCCAATGTATCCGTTGTG3′, **R**-5′TGATTTCCCGTAGGACCGAT3′

Real-time PCR was carried out in a total volume of 25 μL containing 8 ng cDNA template, 0.3 μM of the appropriate primers and the IQ^TM^ SYBR^®^ Green Supermix 1x (Biorad catalog no. 1708885). The PCR protocol consisted of 40 amplification cycles (95 °C for 30 s, 60 °C for 45 s and 79 °C for 15 s) and was performed using an iCycler IQ^TM^ multicolor Real-Time PCR detection system (Bio-Rad). For quantification, a relative standard curve was established for each target gene using four-fold serial dilutions (from 100 to 0.097 ng) of a cDNA template mix prepared using the same conditions. Each sample was normalized to relative expression levels of GAPDH. Calculation of Ct, standard curve preparation and quantification of mRNAs contained in the samples were performed using the “post run data analysis” software provided with the iCycler system (Bio-Rad).

### Cytosolic free Ca^2+^ measurement in single neurons

For cytosolic free Ca^2+^ measurements, all recordings were carried out at 37 °C in Krebs-HEPES buffer (see below) containing 10 µM CNQX (Tocris, Bristol, UK, catalog no. 1045). Acute treatment of neurons with 100 µM GABA (Sigma-Aldrich; St-Louis, MO, USA, catalog no. A2129), 10 µM baclofen (Tocris, catalog no. 0417), 30 µM isoguvacine (Sigma-Aldrich, catalog no. G002) and 10 µM nimodipine (Tocris, catalog no. 0600) was performed in the incubation chamber. Neurons were plated at a density of 1.8 10^5^ cells/cm^2^ on pre-coated 15 mm round glass coverslips with 10 µg/ml poly-L-lysine in phosphate buffered saline. Seven to eleven days after adenoviral infection, neurons were incubated in the dark in the presence of the Ca^2+^ indicator fura-2 acetoxymethylester (Fura-2 AM; catalog no. F1225) at a final concentration of 2 μM in Krebs-HEPES buffer (10 mM HEPES, 135 mM NaCl, 6 mM KCl, 2 mM CaCl_2_, 1.2 mM MgCl_2_, 10 mM glucose, pH 7.4) for 30 min at room temperature. Coverslips were then washed and mounted in a heated (37 °C) microscope chamber (1 ml). Cells were alternately excited (1 or 2 Hz) at 340 and 380 nm for 100 ms using a Lambda DG-4 Ultra High Speed Wavelength Switcher (Sutter Instrument, Novato, CA) coupled to a Zeiss Axiovert 200 M inverted microscope (X20 fluorescence objective) (Zeiss Belgium, Zaventem, BE). Images were acquired using a Zeiss Axiocam camera coupled to a 510 nm emission filter and analysed with the Axiovision software. A total of 70–80 neurons was studied in each experiment and non-neuronal cells were excluded from the analysis as previously described by Pickering and coworkers^80^. Changes in intracellular Ca^2+^ fluorescence were estimated from fluorescence emission intensity ratios F340/F380 or ΔF obtained after excitation of cells at wavelengths of 340 nm and 380 nm. These changes were expressed as ΔF/F0 where F0 is the basal fluorescence value corresponding to the mean of ΔF signals measured during a period of 50 s in control conditions (prior to the GABA pulse). The GABA response was defined as a change of ΔF greater than 10% relative to F0. To estimate the amplitude of the response to GABA, F0 was subtracted from the maximum ΔF value occurring during the 50 s GABA pulse.

### Electrophysiology

In order to preserve intracellular Cl^−^ concentrations, gramicidin perforated patch recordings were performed to measure evoked action potentials in primary cortical cell cultures expressing hAPP between 13 DIV and 19 DIV. The recording bath solution contained 145 mM NaCl, 3 mM KCl, 2 mM CaCl_2_, 2 mM MgCl_2_, 15 mM HEPES and 10 mM glucose (pH adjusted to 7.4 with NaOH, osmolarity: 290 mOsm/l). The internal pipette solution contained 140 mM KCl, 1 mM MgCl_2_, 4 mM MgATP, 0.5 mM EGTA and 10 mM HEPES (pH adjusted to 7.2 with KOH, osmolarity: 280 mOsm/l). A 20 mg/ml stock of gramicidin in dimethyl-sulphoxide (DMSO) was diluted freshly to a final concentration of 80 µg/ml in the internal pipette solution. To facilitate gigaseal formation, patch pipettes were front filled by dipping the tip into the gramicidin-free solution then back filled with the gramicidin containing solution. All recordings were performed at room temperature. To obtain the frequency-current (f-I) relationship, action potential firing was recorded in response to 10 depolarizing current steps of 20 pA using the Axopatch 200B amplifier (Molecular Devices, Sunnyvale, CA, USA) in presence or absence of added GABA (100 µM) (Sigma-Aldrich, St-Louis, MO, USA, catalog no. A2129). Data were filtered at 5 kHz through a lowpass Bessel filter and collected at a frequency of 8 kHz using the Digidata 1322 A digitizer (Molecular Devices, Sunnyvale, CA, USA). Liquid junction potential and capacitance transients were compensated. 20 to 30 min after cell-attached seal formation (seal resistance >3 GΩ), series resistance (Rs) typically dropped and stabilized at ~30–40 MΩ. Resting membrane potential measured in current clamp mode (I = 0) was stable. Input and series resistance were monitored during the experiment, and recordings were excluded when any of these parameters changed by >10%. The instantaneous firing rate for each current step was calculated from the inter-spike interval using the pClamp software. Electrodes were pulled from capillary glass (Harvard Apparatus, Les Ulis Cedex, France, catalog no. 30-0053) on a Sutter Instruments P97 puller (Sutter Instruments, Novato, CA, USA) and had a resistance of 3–6 MΩ when filled with the internal solution.

### Statistics

Statistical analyses were performed using GraphPad Prism 7.01. The Shapiro-Wilk test was used to test for the normality of data. Parametric testing procedures (Student’s *t* test or one-way analysis of variance (ANOVA) followed by Bonferroni’s multiple-comparison post-test when many subgroups were compared) were applied for normally distributed data, otherwise nonparametric tests were used (Mann-Whitney or Kruskal-Wallis tests followed by Dunn’s multiple-comparison post-test when many subgroups were compared). The total number of samples (n) analysed in all experimental conditions (number of repeated measurements) is indicated in figures legends. Results are presented as mean ± s.e.m. and statistical significance was set at *P* values (two-tailed tests) <0.05 (*P < 0.05, **P < 0.01; ***P < 0.001).

## Electronic supplementary material

Supplementary Information
