# Supplementary material for: Cortical cells reveal APP as a new player in the regulation of GABAergic neurotransmission
Source: Sci Rep. 2017 Mar 23;7:370. doi: 10.1038/s41598-017-00325-2 (PMC5428293; doi:10.1038/s41598-017-00325-2)
Supplement: Supplementary file 1 — Supplementary Information [file 41598_2017_325_MOESM1_ESM.pdf]

Title:

Cortical cells reveal APP as a new player in the regulation of GABAergic neurotransmission

Authors and their affiliations:

Anna Doshina[1], Florian Gourgue[1], Michiho Onizuka[1], Remi Opsomer[1], Peng Wang[1], Kunie Roussel-Ando[2], Bernadette Tasiaux[1], Ilse Dewachter[1], Pascal Kienlen-Campard[1], Jean-Pierre Brion[2], Philippe Gailly[1], Jean-Noël Octave[1*] and Nathalie Pierrot[1].

[1] Institute of Neuroscience, Université catholique de Louvain, 1200 Brussels, Belgium,

[2] Laboratory of Histology and Neuropathology, Université libre de Bruxelles, 1070 Brussels, Belgium.

[*]Correspondence: Jean-Noël Octave (email : jean-noel.octave@uclouvain.be)

Supplementary information:

- **Supplementary methods**
- **Supplementary Figures 1-13**
- **Supplementary table 1**
- **Supplementary references**
- **Supplementary Figure 14 (all full and un-cropped gels)**

Supplementary methods

Human brain tissues

Frontal cortex brain tissue samples from 9 human control subjects and 9 demented patients were collected with the approval of the Ethical Committee of the Medical School of the Université Libre de Bruxelles or via the rapid autopsy program of the Netherlands Brain Bank (NBB), Amsterdam, which provides postmortem specimens from clinically well documented and neuropathologically confirmed cases. The work of the NBB abides by the ethical code of conduct approved by the ethics committee. Ethical approval and written informed consent from the donors or the next of kin were obtained in all cases. All patients were clinically diagnosed as sporadic cases of Alzheimer's disease (AD). Supplementary table 1 summarizes the clinical data and the neuropathological staging of control subjects and patients with AD according to Braak and Braak staging for neurofibrillary tangles[1] and the Consortium to Establish a Registry for AD score for senile plaques[2].

Culture reagents and antibodies

Mouse monoclonal Tuj 1 (Neuron-specific class III β-tubulin; Neuromics, Edina, MN, USA, catalog no. MO 15013). Anti-MAP2 and rabbit polyclonal actin (Sigma-Aldrich, St-Louis, MO, USA, catalog nos. M4403 and A2066, respectively).

Aβ measurements

Human Aβ1-40 levels were measured in cell culture supernatants using the Multi-Spot Human (6E10) Aβ Triplex Assay and the SECTOR Imager 2400 (MesoScale Discovery, Rockville, MD, USA, catalog no. K15200E-1) according to manufacturer's instructions.

Cytotoxicity assay

Cell viability was measured by LDH release in the culture medium at 13 DIV using Cytotoxicity Detection kit (Sigma-Aldrich, St-Louis, MO, USA, catalog no. 11644793001) according to the manufacturer's instructions. Relative absorbance was measured at 490 nm using a VICTOR Multilabel Plate Reader (PerkinElmer, Richmond, VA, USA). Background LDH release was determined in non-infected control cultures.

Western blotting for human brain tissues

Anti-βIII tubulin and anti-actin were used both at 1:1 000.

Immunofluorescence

Cells were seeded at 10^5 cells/cm^2 on glass coverslips, fixed with 4% v/v formaldehyde at room temperature (RT) then washed in PBS and permeabilized 1 h with 0,4% Triton X100 (v/v) in PBS containing bovine serum albumin (Sigma-Aldrich, St-Louis, MO, USA, catalog no. A7906) 3%. After three washes in PBS cells were incubated 1h at RT with primary antibodies: WO2 (1:1 000); anti-KCC2 (1:100), MAP2 (1:1 000). After three PBS washes, cells were incubated for 1h with 5 µg/ml Alexa-labelled secondary antibodies in presence of Hoechst (1:10 000, catalog no. 62249). After three additional PBS washes, preparations were mounted in Fluoprep (Biomérieux, Marcy l'Etoile, FR, catalog no. 75521). Pictures were acquired with an AMG Evos fluorescence digital inverted microscope (Advanced Microscopy Group, Mill Creek, WA, USA). Immunoreactivity was quantified on 20x digital images by using Image J software with thresholds set according to signal intensity[3].

Supplementary Figures 1-13

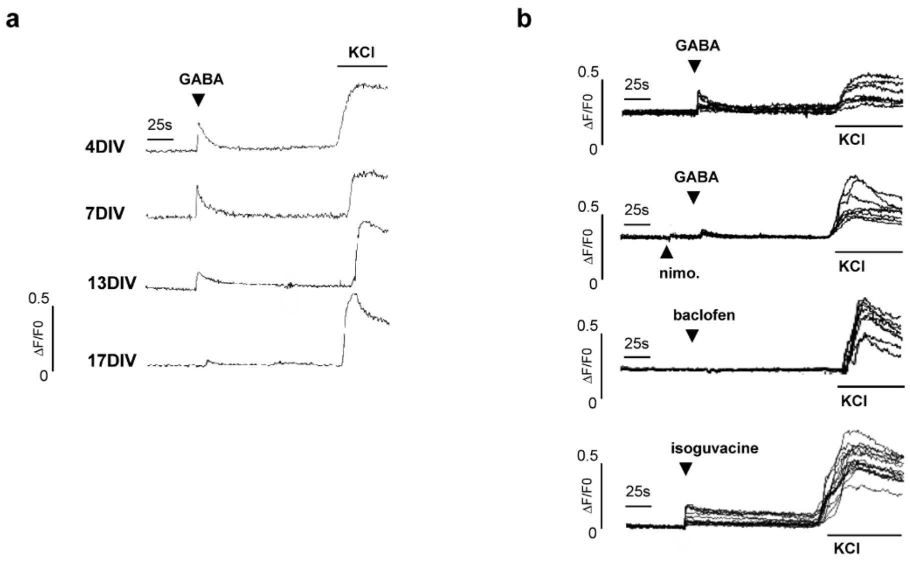

Supplementary Figure 1. **Characterization of GABA-induced depolarization in rat cortical cells cultures**. (**a**) Time course of GABA-induced calcium fluorescence in cortical cells cultures between 4 and 17 days in vitro (DIV). One representative recording of a cell baring a $[Ca2^{+}]_i$ increase in response to 100 µM GABA at each time point. High concentration of extracellular KCl (50 mM) was applied at the end of the recording. (**b**) Comparative recordings of GABA-induced Ca^{2+} increase in rat cortical cultures at 13 DIV (GABA 100 µM). Representative traces of ten cells treated with GABA in extracellular solution containing 10 µM L-type VDCC antagonist nimodipine (middle panel), 10 µM $GABA_B$ agonist baclofen or 30 µM $GABA_A$ agonist isoguvacine (lower panels). All traces are expressed as ΔF/F0, were ΔF is a change in the F340/F380 ratio and F0 is the basal fluorescence value.

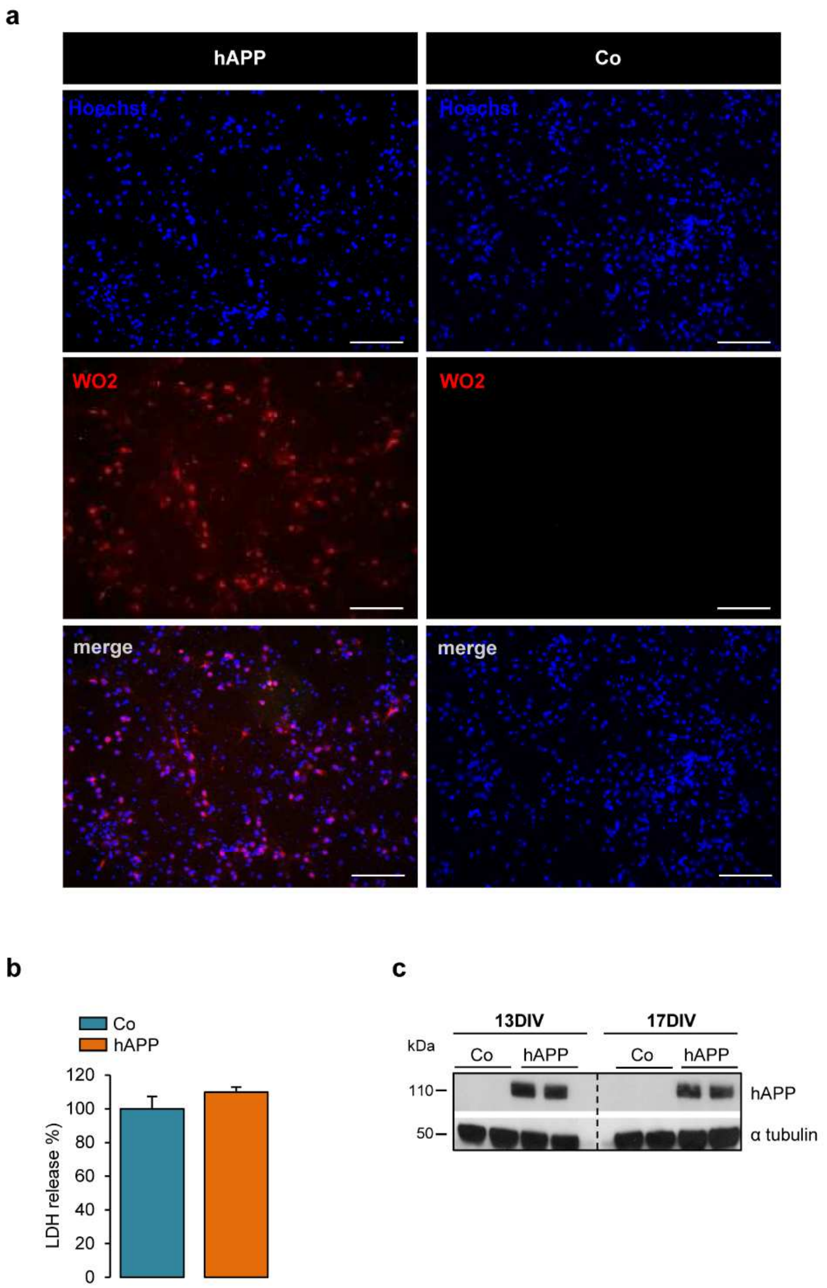

Supplementary Figure 2. **Homogeneous, stable and not toxic expression of hAPP**. (**a**) Representative immunofluorescent staining of human APP (hAPP) expression in primary rat cortical cultures infected or not (control, Co) at 6 DIV with an adenovirus encoding hAPP. At 13 DIV, hAPP was labelled with the specific WO2 antibody recognizing the 4-10 amino-acids of the human Aβ sequence. Scale bar, 60 µm. (**b**) Measurement of LDH activity released after hAPP-adenoviral transduction at 13 DIV in primary rat cortical cultures. Background LDH release was determined in non-infected control cultures (Co). Results were expressed as

percentage of total LDH release in non-infected control cultures (Co), (Co=12, hAPP=14 analysed in 2 independent experiments, Co vs hAPP $P>0.05$, Student's *t*-test, non-significant (n.s.). (**c**) Representative Western blot of cell lysates from Co and hAPP expressing cortical cultures analysed at 13 and 17 DIV.

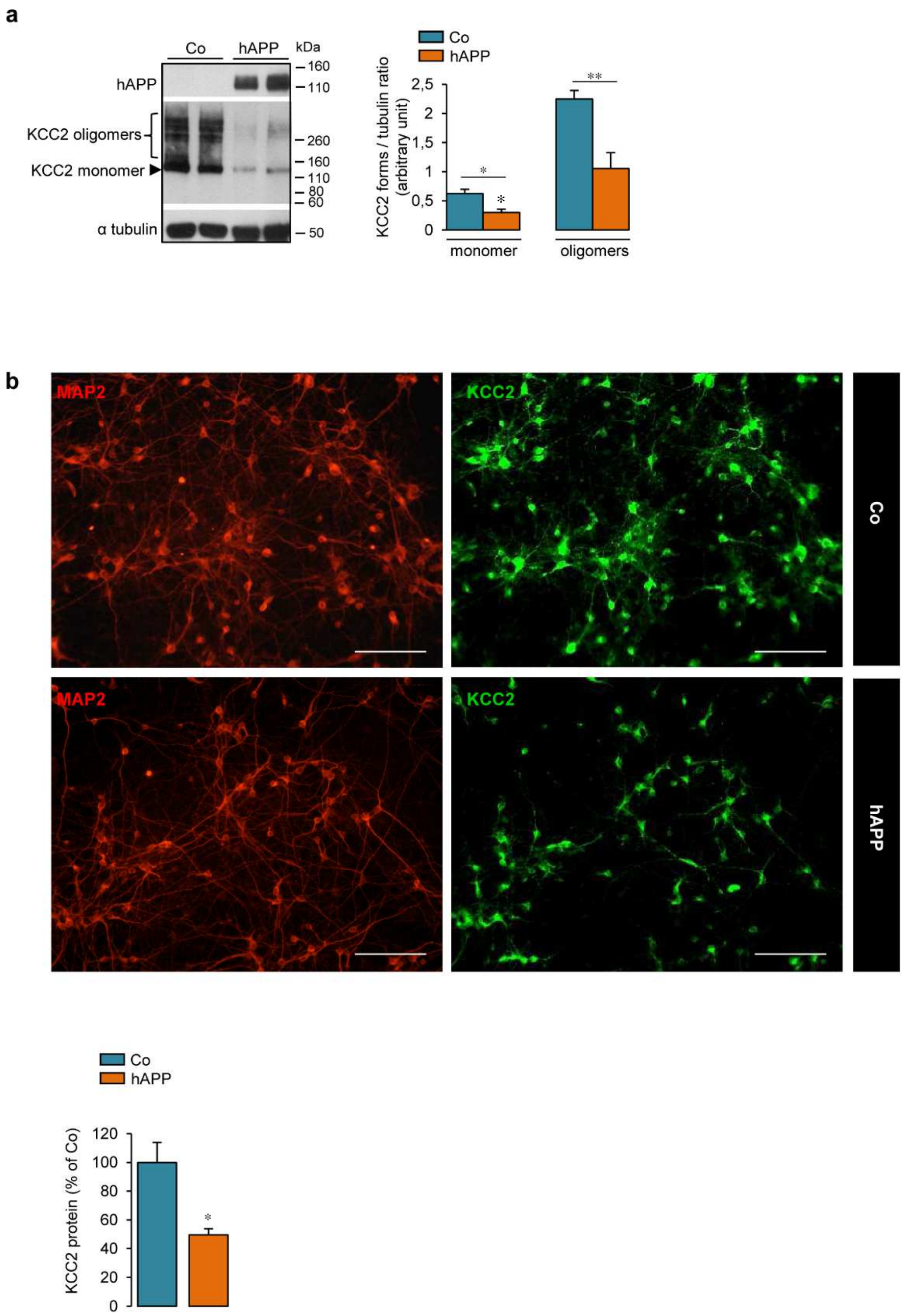

Supplementary Figure 3. **hAPP expression decreases KCC2 oligomers and KCC2 immunofluorescent signal**. (**a**) Representative Western blot of cell lysates from primary rat cortical cultures infected or not (control, Co) at 6 DIV with an adenovirus encoding human APP (hAPP) showing monomeric and oligomeric forms of KCC2 at 17 DIV. Blots were probed

using anti-KCC2 and -α tubulin antibodies. Right panel: quantification of monomeric or oligomeric KCC2 (mean ± s.e.m.) / α-tubulin ratios (n=8 of each analysed in 3 independent experiments, ∗*P*<0.05, ∗∗*P*<0.01, Mann-Whitney test). (**b**) Representative immunofluorescent staining and quantification of KCC2 expression in Co and hAPP expressing cortical cultures. Bottom panel: quantification of KCC2 immunofluorescent signal. Data are expressed as percentage (mean ± s.e.m.) of Co (n=6 of each analysed in 3 independent experiments, ∗*P*<0.05, Student's *t*-test). Scale bar, 60 µm..

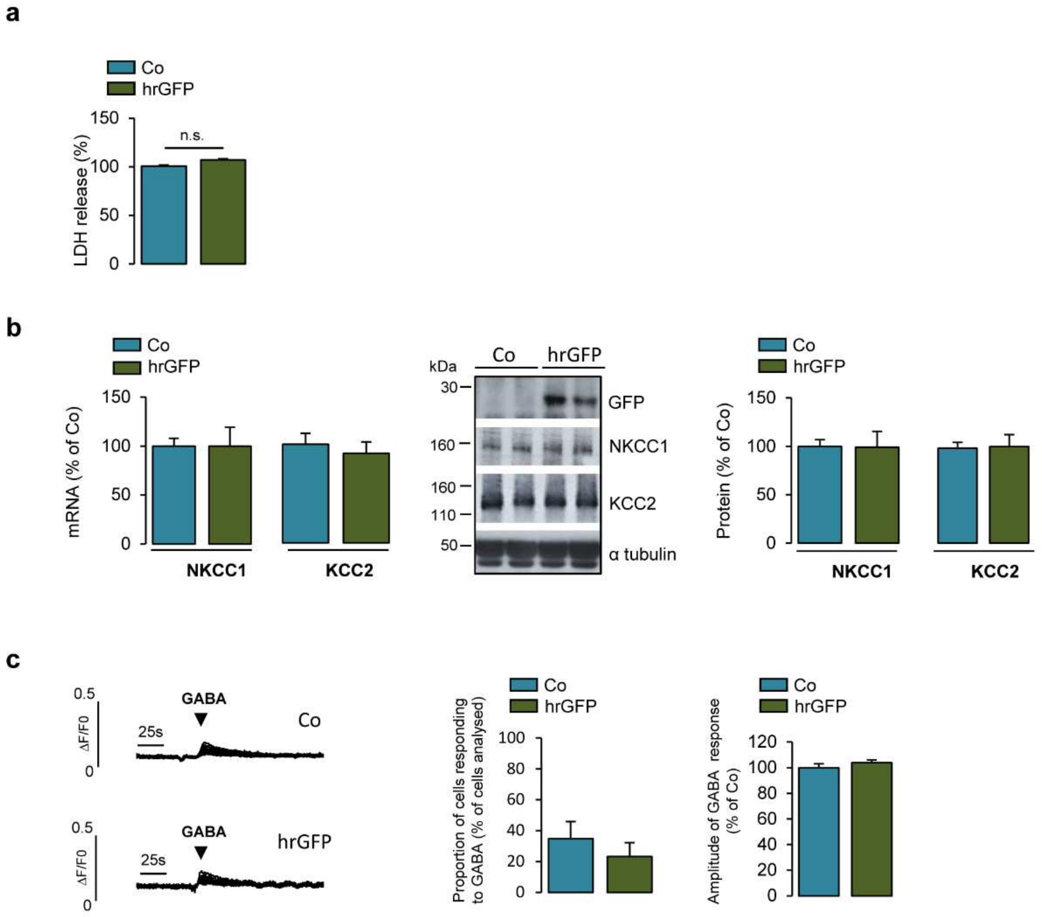

Supplementary Figure 4. **Unaffected KCC2 expression GABA responses in hrGFP expressing cultures**. (**a**) Measurement of LDH activity released from primary rat cortical cultures infected or not (control, Co) at 6 DIV with an adenovirus encoding human recombinant GFP (hrGFP). Results were obtained at 13 DIV and expressed as percentage of total LDH release in Co cultures. Co=12, hrGFP=14 in 2 independent experiments, Co vs hrGFP $P>0.05$, Student's *t*-test, non-significant (n.s.). (**b**) Left panel: Comparative RT-qPCR analyses of *Slc12a2* (encoding NKCC1) and *Slc12a5* (encoding KCC2) mRNA levels from Co and hrGFP cortical cultures. Results were obtained at 13 DIV, normalized to GAPDH mRNA and expressed as percentage (mean ± s.e.m.) of Co (Co n=10 and hrGFP n=10 in 4 independent experiments, Student's *t*-test). Middle panel: Representative Western blot of 13 DIV cell lysates from Co and hrGFP expressing cortical cultures. Expression of hrGFP was monitored and blots were further probed using anti-NKCC1, -KCC2, and -α tubulin antibodies. Right panel:

quantification of NKCC1 and KCC2 / α tubulin ratios. Results were expressed as percentage (mean ± s.e.m.) of Co (Co n=7 and hrGFP n=4 analysed in 4 independent experiments, $P>0.05$, Student's *t*-test). (**c**) GABA (100μM)-induced calcium fluorescence in Co and hrGFP expressing cortical cultures analysed at 17 DIV. Representative traces of ten cell recordings per condition (total of 213 hrGFP cells analysed in 5 independent experiments). Traces are expressed as ΔF/F0, were ΔF is a change in the F340/F380 ratio and F0 is the basal fluorescence value. Right panel: percentage (mean ± s.e.m.) of cells responding to 100 μM GABA and amplitude of this response (right panels) (Co n=205, hrGFP n=155, cells in 3 independent experiments, $P>0.05$, Student's *t*-test).

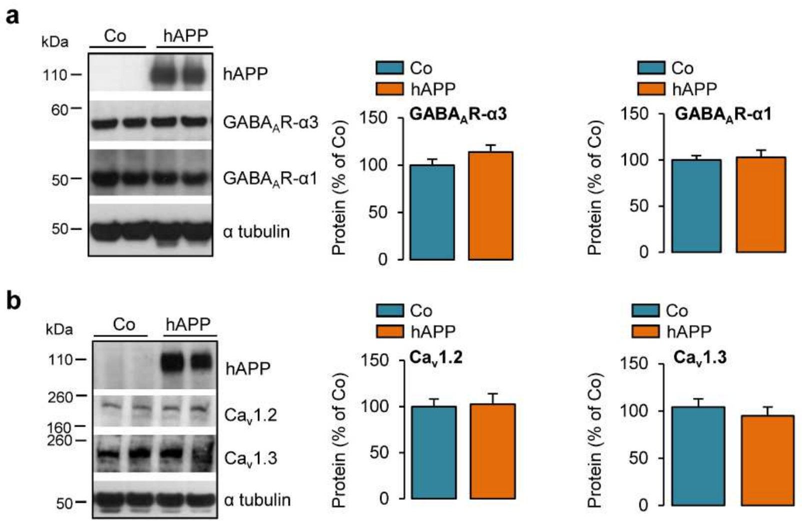

Supplementary Figure 5. **hAPP expression does not modify GABA$_A$R α3/α1 and L-type VDCC α1C/D pore-forming subunits expression**. (**a** and **b**) Representative Western blots of cell lysates from primary rat cortical cultures infected or not (control, Co) at 6 DIV by an adenovirus encoding human APP (hAPP). Results were obtained between 13 and 17 DIV. Expression of hAPP was monitored with the specific WO2 antibody. Anti-GABA$_A$R antibodies were used to detect GABA$_A$R-α1 and α3 subunits and anti-CaV1.3 and -CACNA1C to detect α1D and α1C pore-forming subunits of L-type VDCC (Ca$_V$1.2 and Ca$_V$1.3, respectively). Blots were further probed using anti-α tubulin antibody. Right panels: quantification of GABA$_A$R-α3, -α1, Ca$_V$1.2 and Ca$_V$1.3 / α tubulin ratios. Results were expressed as percentage (mean ± s.e.m.) of Co ((**a**) n=18 of each analysed in 8 independent experiments and (**b**) n=11 of each analysed in 5 independent experiments, *P*>0.05, Student's *t*-test).

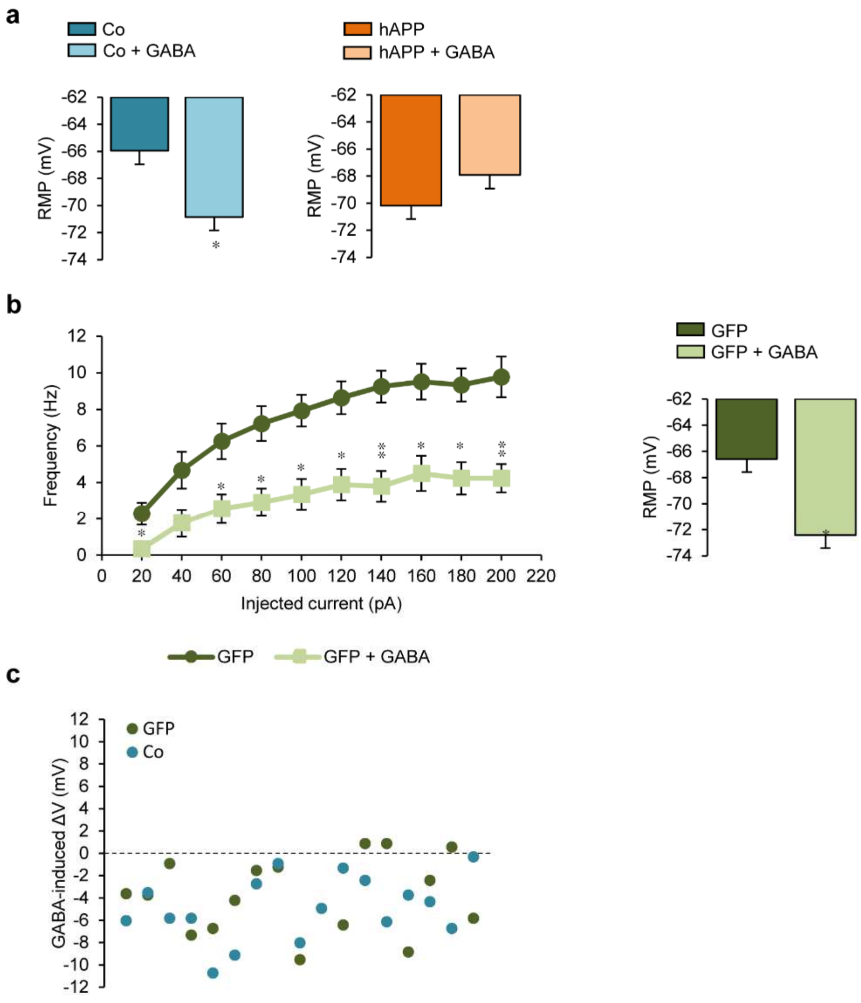

Supplementary Figure 6. **Electrophysiological responses to GABA are not affected in hrGFP expressing cultures**. (**a**) Resting membrane potential (RMP) measured between 13 and 19 DIV after a bath application of GABA (100µM) in primary rat cortical cultures infected or not (control, Co) at 6 DIV by an adenovirus encoding human APP (hAPP) (Co n=17 and hAPP n=19 cells analysed in 6 independent experiments, *$P<0.05$, Student's *t*-test). (**b**) Frequency of action potentials depending on the current injected in human recombinant GFP (hrGFP) expressing cortical neurons before or after a bath application of GABA (100µM) measured between 13 and 19 DIV (*$P<0.05$, **$P<0.01$ Student's *t*-test applied for each current step to compare the effect of GABA on hrGFP cultures) Right panel: RMP measured between 13 and 19 DIV in Co and hrGFP expressing cortical cultures. (**c**) Difference in membrane potential

(ΔV) elicited by a bath application of GABA (100µM) measured in hrGFP expressing cortical cultures between 13 and 19 DIV (hrGFP n=18 cells analysed in 4 independent experiments).

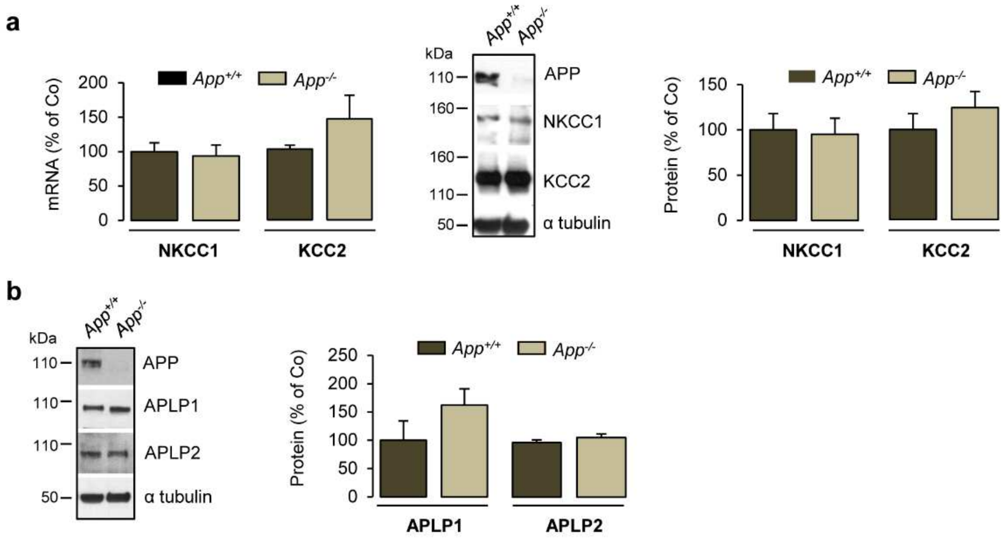

Supplementary Figure 7. **Unaffected expression of NKCC1 and KCC2 in *App* knock-out cells and APLP1 and 2 expression**. (**a**) Comparative RT-qPCR analyses of *Slc12a2* (encoding NKCC1) and *Slc12a5* (encoding KCC2) mRNA levels from primary cultures of mouse cortical cells of wild type (*App*$^{+/+}$) and *App* knockout mice *(App*$^{-/-}$*)*. Results were obtained at 13 DIV, normalized to GAPDH mRNA and expressed as percentage (mean ± s.e.m.) of *App*$^{+/+}$ (n=10 of each analysed in 8 independent experiments, P>0.05, NKCC1: Student's *t*-test and KCC2: Mann-Whitney test). (**a** (Middle panel) and **b**) Representative Western blots of cell lysates from *App*$^{+/+}$ and *App*$^{-/-}$ cortical cultures at 13 DIV. Expression of endogenous APP (APP) was monitored with the anti-APP C-terminal antibody. Blots were further probed with anti-APLP1 and 2, anti-NKCC1, anti-KCC2 and anti-α tubulin antibodies. Right panels: quantification of NKCC1 and KCC2 / α tubulin ratios (**a**) or APLP1 and APLP2 / α tubulin ratios (**b**). Results were expressed as percentage (mean ± s.e.m.) of *App*$^{+/+}$ (*App*$^{+/+}$ and *App*$^{-/-}$ n=3 of each analysed in 3 independent experiments), P>0.05, Student's *t*-test.

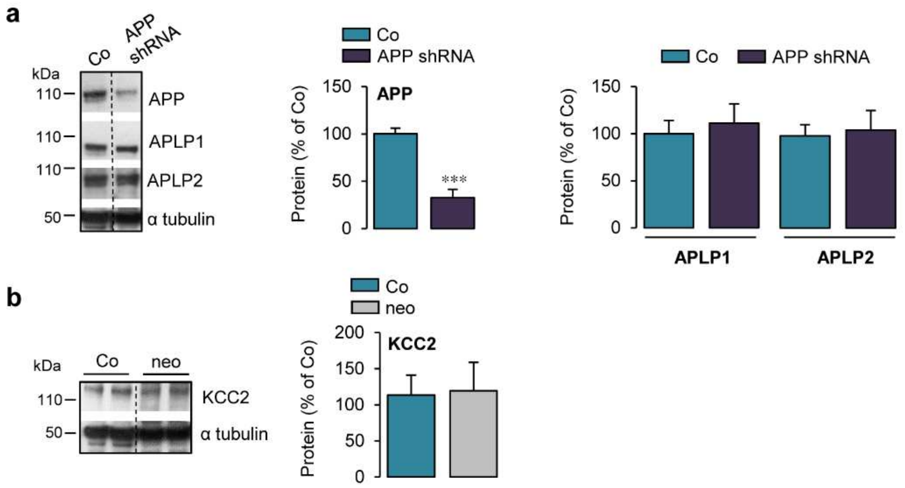

Supplementary Figure 8. **Unaffected expression of APLP1 and APLP2 in APP knock-down cultures and KCC2 expression in neomycin infected control cultures**. (**a** and **b**) Left panels: Representative Western blots of cell lysates from primary rat cortical cultures infected or not (control, Co) at 6 DIV with recombinant lentiviruses encoding APP shRNA or the neomycin resistance gene (neo) (**a** and **b** respectively). Results were obtained between 13 DIV and 17 DIV. Expression of endogenous APP (APP) was monitored with the anti-APP C-terminal antibody. Blots were further probed with anti-APLP1 and 2, anti-KCC2 and anti-α tubulin antibodies. Right panels: quantification of APP, APLP1, APLP2 and endogenous APP / α tubulin ratios or KCC2 / α tubulin ratios (**a** and **b**, respectively). Results were expressed as percentage (mean ± s.e.m.) of Co (Co and APPshRNA n=6, neo n=3 in 3 independent experiments), $P>0.05$, ***$P< 0.001$, Student's t-test.

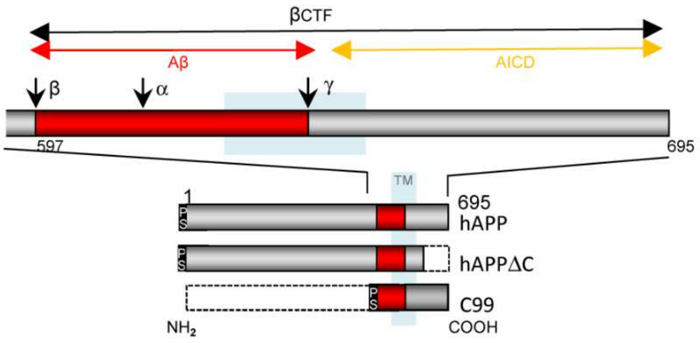

Supplementary Figure 9. **APP proteolytic fragments.** Schematic representation of hAPP metabolites and constructs used in this study. hAPP (695 amino acids) is a transmembrane (TM, blue) protein with one membrane spanning domain processed by α-, β- and γ-secretase cleavages to generate β-cleaved carboxyl terminal fragment (βCTF, residue 597 to residue 695 corresponding to the last 99 amino acids of hAPP known as C99), Aβ peptide (red box) and the APP intracellular domain (AICD, yellow). hAPPΔC - hAPP695 truncated in its C-terminal region from residue 652 to residue 695. SP, signal peptide.

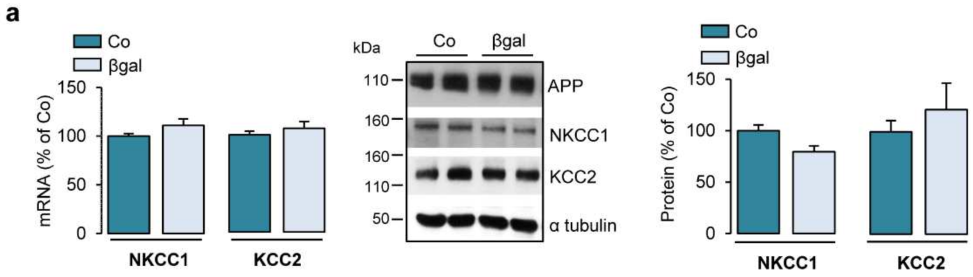

Supplementary Figure 10. **Unaffected expression of NKCC1 and KCC2 in βgal infected control cortical cultures**. Left panel: Comparative RT-qPCR analyses of *Slc12a2* (encoding NKCC1) and *Slc12a5* (encoding KCC2) mRNA levels in primary rat cortical cultures infected or not (control, Co) at 6 DIV with an adenovirus encoding β-galactosidase (βgal). Results were obtained between 13 and 17 DIV, normalized to GAPDH mRNA and expressed as percentage (mean ± s.e.m.) of Co (Co n=11 and βgal n=7 analysed in 5 independent experiments); $P>0.05$, Student's *t*-test. Middle panel: Representative Western blot of cell lysates from βgal cortical cultures. Expression of endogenous APP (APP) was monitored with the anti-APP C-terminal antibody. Blots were further probed with anti-NKCC1, anti-KCC2 and anti-α tubulin antibodies. Right panel: quantification of NKCC1 and KCC2 / α tubulin ratios. Results were expressed as percentage (mean ± s.e.m.) of Co (Co and βgal n=7 of each analysed in 5 independent experiments), $P>0.05$, Student's *t*-test - except for KCC2 a Mann-Whitney test was applied.

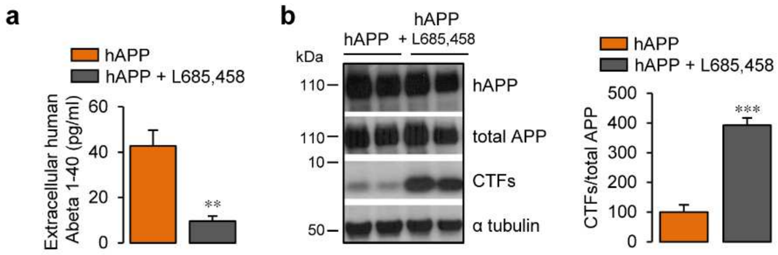

Supplementary Figure 11. **Inhibition of γ-secretase activity with L685,458**. (**a**) Effect of 10 µM L685,458 / 24 h, a γ-secretase inhibitor, on human extracellular Aβ1-40 production (pg/ml) at 13 DIV in primary rat cortical cultures infected with an adenovirus encoding human APP (hAPP) (n=9 of each in 3 independent experiments, ***P*< 0.01, Mann-Whitney test). (**b**) Left panel: Representative Western blot of cells lysates from 13 DIV hAPP expressing cortical cultures treated or not with 10 µM L685,458/24h. Expression of hAPP was monitored with the specific WO2 antibody and expression of total APP and APP carboxyl terminal fragments (CTFs) was monitored with the anti-APP C-terminal antibody. Right panel: quantification CTFs / α tubulin ratios. Results were expressed as percentage (mean ± s.e.m.) of untreated hAPP expressing cells (n=9 of each in 4 independent experiments, ****P*< 0.001, Student's *t*-test).

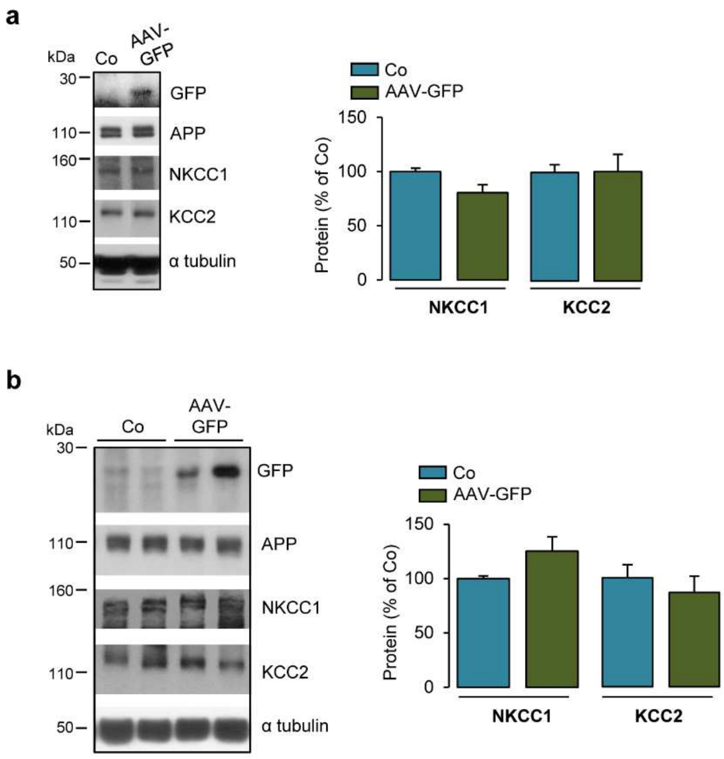

Supplementary Figure 12. **AAV-GFP transduction *in vitro* and *in vivo*.** (**a**) Representative Western blot of cell lysates from primary rat cortical cultures infected or not (control, Co) at 6 DIV with serotype 2 associated-adenoviruses encoding green fluorescent protein (AAV-GFP). Results were obtained at 13 DIV. Expression of endogenous APP (APP) was monitored with the anti-APP C-terminal antibody. Blots were further probed using anti-NKCC1, -KCC2, GFP and -α tubulin antibodies. Right panel: quantification of NKCC1 and KCC2 / α tubulin ratios. Results were expressed as percentage (mean ± s.e.m.) of Co (n=3 of each analysed in 3 independent experiments, P>0.05, Student's t-test except for KCC2 a Mann-Whitney test was applied). (**b**) Representative Western blot of brain homogenates from mice intraventricularly injected at P1-P2 with AAV-GFP. 30 days post injection, expression of GFP was monitored with the anti-GFP antibody and endogenous APP (APP) with the anti-APP C-terminal antibody. Blots were further probed using anti-NKCC1, -KCC2, and -α tubulin antibodies. Right panel: NKCC1 and KCC2 / α tubulin ratios were quantified. Results were expressed as percentage

(mean ± s.e.m.) of Co (NKCC1, Mann-Whitney test and KCC2 Student's *t*-test. Co n=7 mice and AAV-GFP n=8 mice).

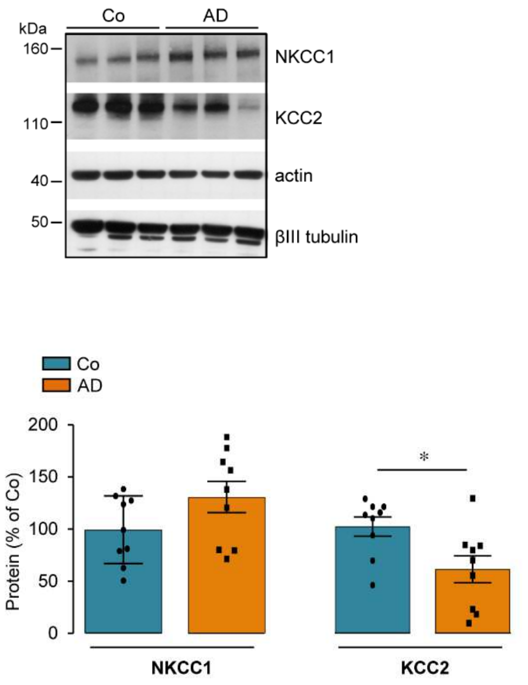

Supplementary Figure 13. **Expression of NKCC1 and KCC2 in late onset AD brains.** Representative Western blot of NKCC1 and KCC2 expression in frontal cortex of postmortem human brain tissues from control subjects (Co, n=9) and patients with Alzheimer disease (AD, n=9). Blots were probed using anti-NKCC1, -KCC2, -actin and -βIII tubulin antibodies. Bottom panel: quantification of NKCC1 / actin and KCC2 / βIII tubulin ratios. Results were expressed as percentage (mean ± s.e.m.) of Co, $*P<0.05$, Student's *t*-test.

	Case number	Age at death (years)	Sex	Post-mortem interval (h)	Braak stageing (NFT)	CERAD Plaque score (amyloid)
**Control subjects**						
	1	78	M	23	0	0
	2	69	M	6	0	0
	3	58	M	5.5	I-II	0
	4	92	F	NA	0	0
	5	60	F	28	0	0
	6	79	M	6.5	I	A
	7	70	F	7.5	II	A
	8	79	M	6	II	A
	9	78	F	7	I	A
**AD patients**						
	1	72	M	44	VI	C
	2	76	M	10	VI	C
	3	60	M	37	VI	C
	4	78	F	24	VI	C
	5	79	F	25	VI	C
	6	83	F	23	VI	C
	7	73	F	6	VI	C
	8	77	M	6	VI	C
	9	74	M	7.5	VI	C

	**Post-mortem interval** (h) Mean ± SEM	**Age at death** (years) Mean ± SEM	**Sex F/M**
**Control subjects**	11.19 ± 3.16	73.67 ± 3.53	4/5
**AD patients**	20.28 ± 4.65[a]	74.67 ± 2.14[b]	4/5

Supplementary Table 1. **Summary of the characteristics of control subjects and AD patients**. ([a] and [b]) Mean post-mortem interval ([a]) and age ([b]) were not significantly different between control subjects (n=9) and Alzheimer patients (n=9) (P = 0.1368 and P = 0.8119, respectively; Student's *t* test). The neuropathological staging of AD patients is determined according to the Braak and Braak staging and the CERAD plaque score. NA, not-available; NFT, neurofibrillary tangles.

Supplementary references

1. Braak,H. & Braak,E. Neuropathological stageing of Alzheimer-related changes. *Acta Neuropathol. (Berl)* **82**, 239-259 (1991).

2. Mirra,S.S. *et al.* The Consortium to Establish a Registry for Alzheimer's Disease (CERAD). Part II. Standardization of the neuropathologic assessment of Alzheimer's disease. *Neurology* **41**, 479-486 (1991).

3. Jensen,E.C. Quantitative analysis of histological staining and fluorescence using ImageJ. *Anat. Rec. (Hoboken.)* **296**, 378-381 (2013).

Supplementary Figure 14 (all full and un-cropped gels)

Fig. 1a

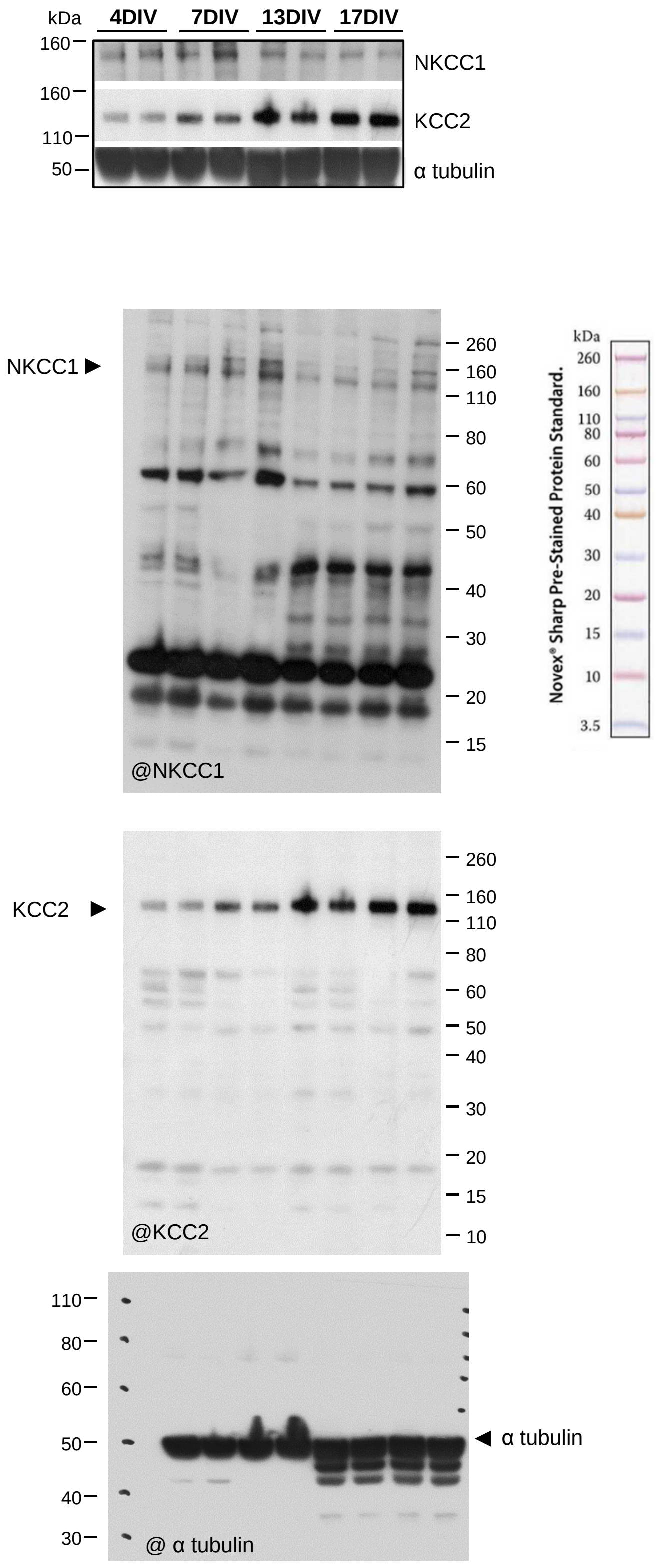

Fig. 2a

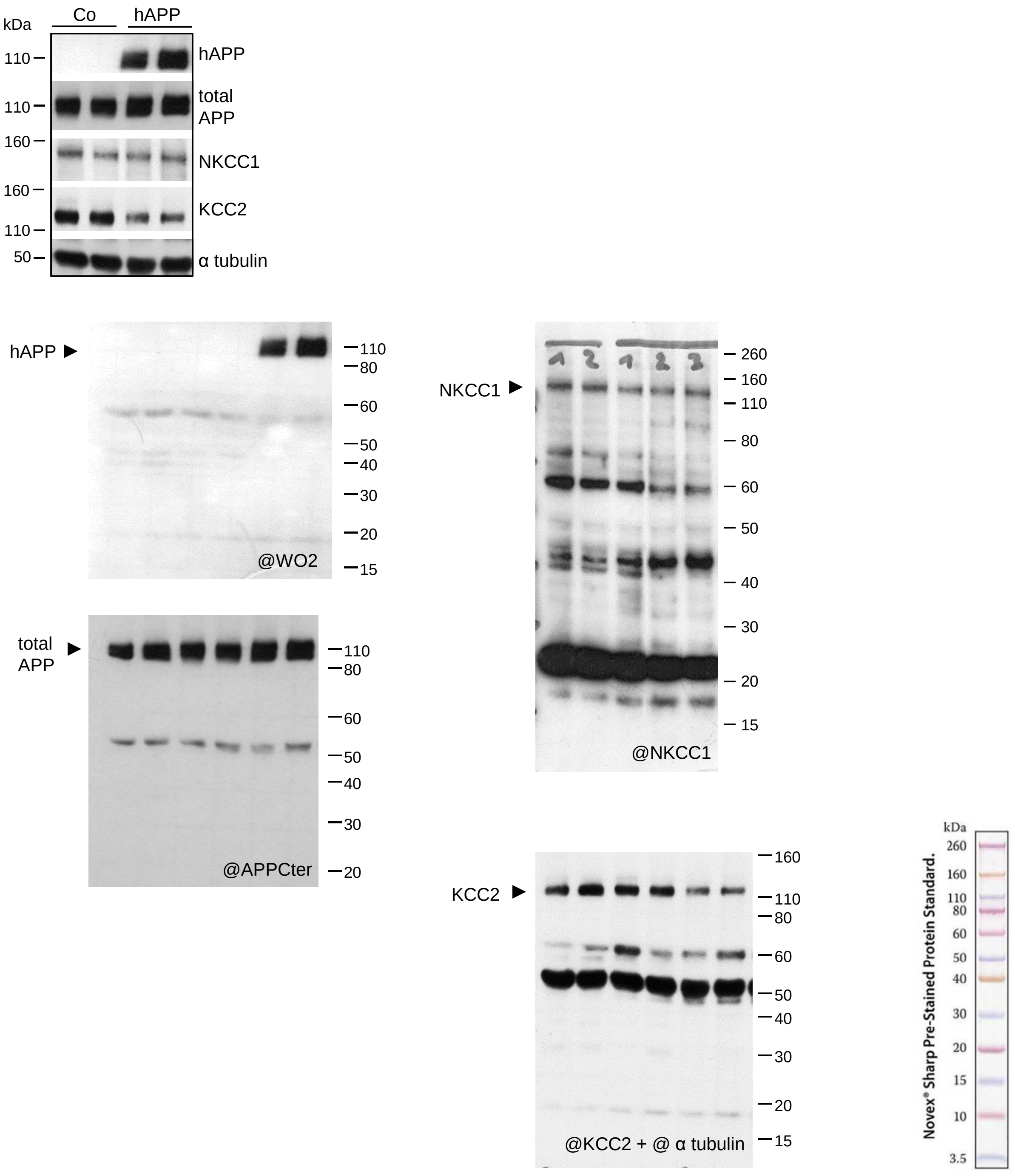
kDa
Co
hAPP
110
hAPP
110
total APP
160
NKCC1
160
KCC2
110
50
α tubulin
hAPP
110
80
60
50
40
30
20
@WO2
15
total APP
110
80
60
50
40
30
@APPCter
20
NKCC1
260
160
110
80
60
50
40
30
20
15
@NKCC1
KCC2
160
110
80
60
50
40
30
20
@KCC2 + @ α tubulin
15
kDa
Novex® Sharp Pre-Stained Protein Standard.
260
160
110
80
60
50
40
30
20
15
10
3.5

Fig. 3a

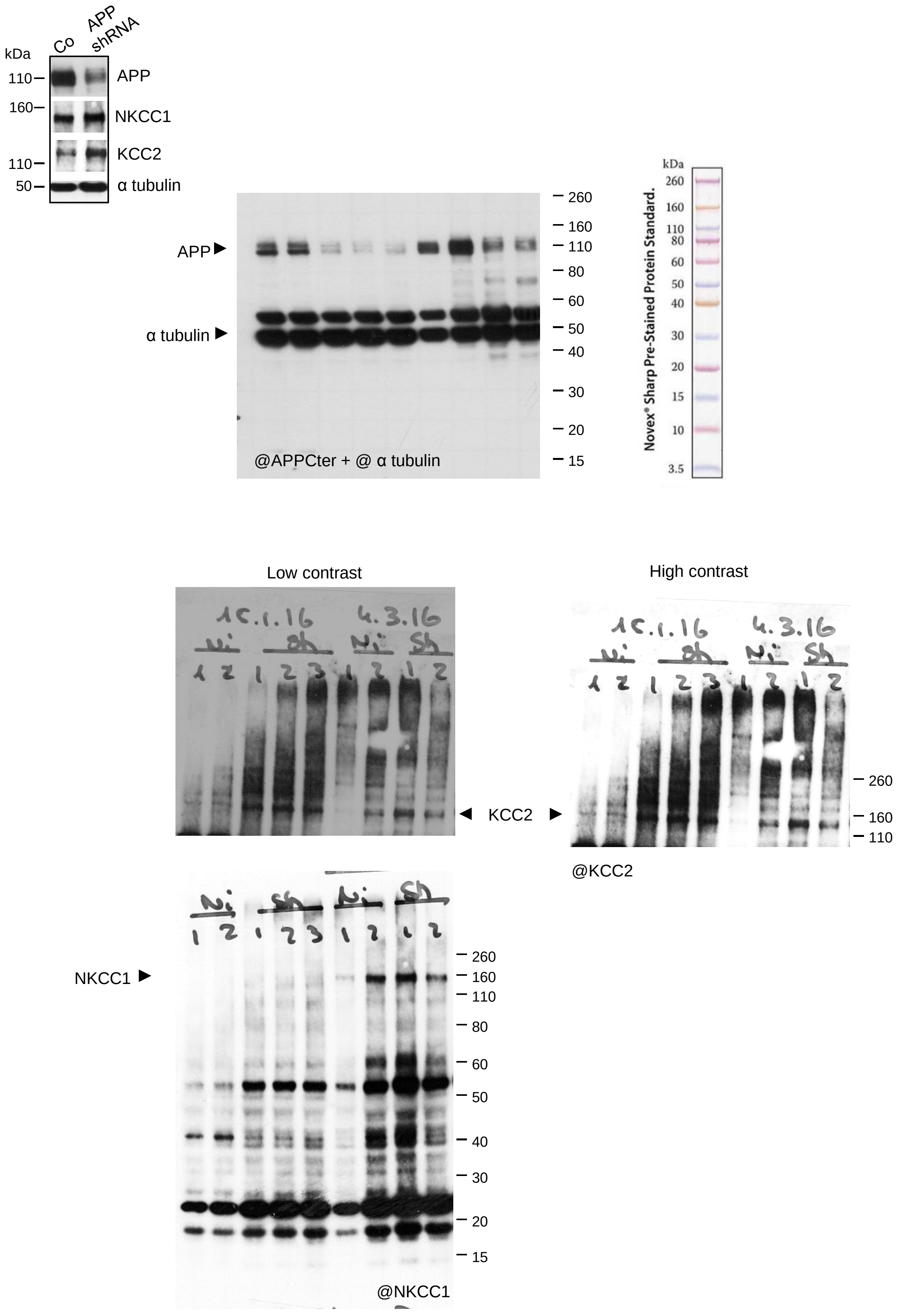

Co
APP shRNA
kDa
110
160
110
50
APP
NKCC1
KCC2
α tubulin
APP
α tubulin
260
160
110
80
60
50
40
30
20
15
@APPCter + @ α tubulin
kDa
Novex® Sharp Pre-Stained Protein Standard.
3.5
10
Low contrast
High contrast
KCC2
@KCC2
NKCC1
@NKCC1

Fig. 3b

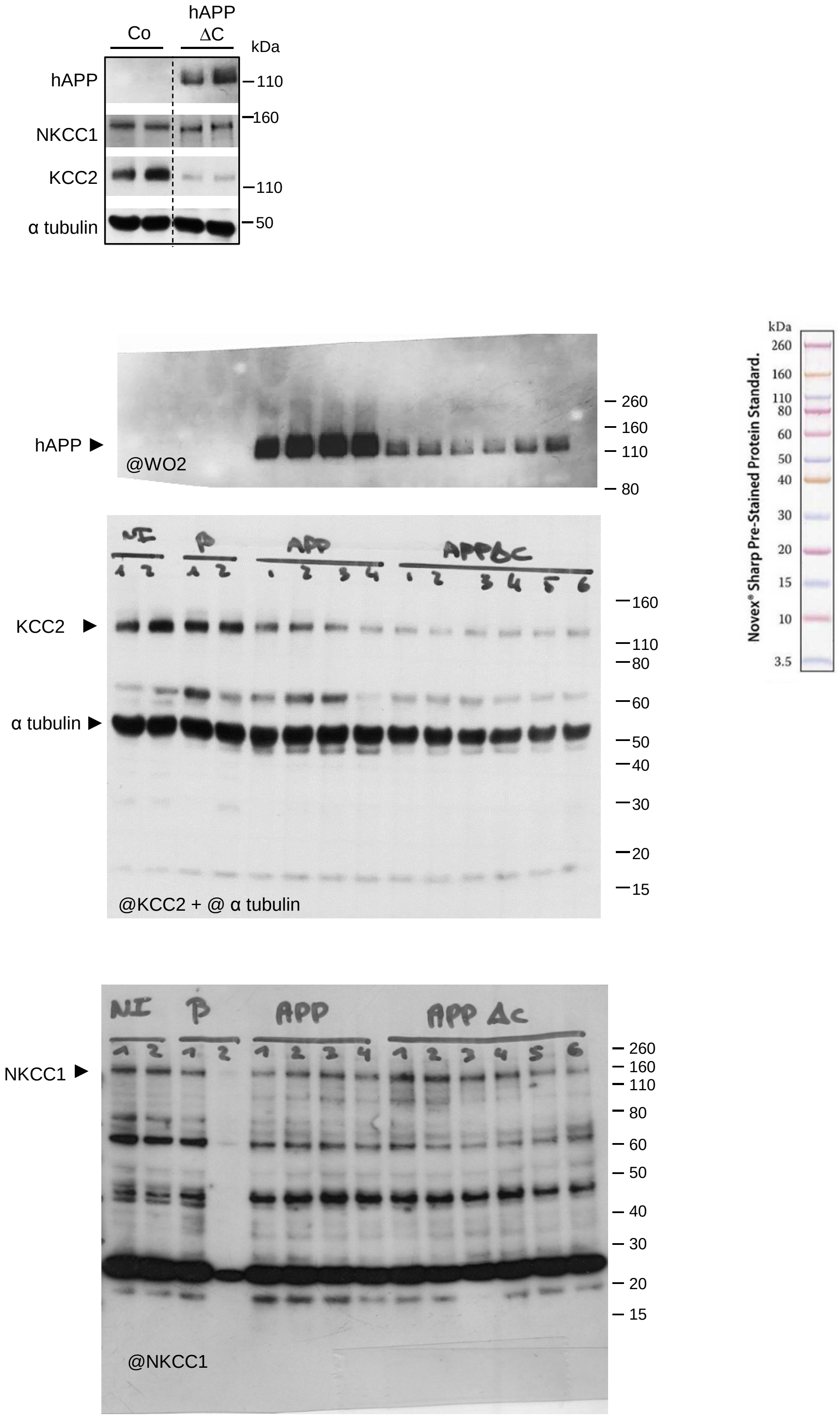

Co
hAPP ΔC
kDa
hAPP
NKCC1
KCC2
α tubulin
110
160
110
50
hAPP ►
@WO2
260
160
110
80
KCC2 ►
α tubulin ►
@KCC2 + @ α tubulin
160
110
80
60
50
40
30
20
15
NKCC1 ►
@NKCC1
260
160
110
80
60
50
40
30
20
15
kDa
Novex® Sharp Pre-Stained Protein Standard.
260
160
110
80
60
50
40
30
20
15
10
3.5

Fig. 3b (continue)

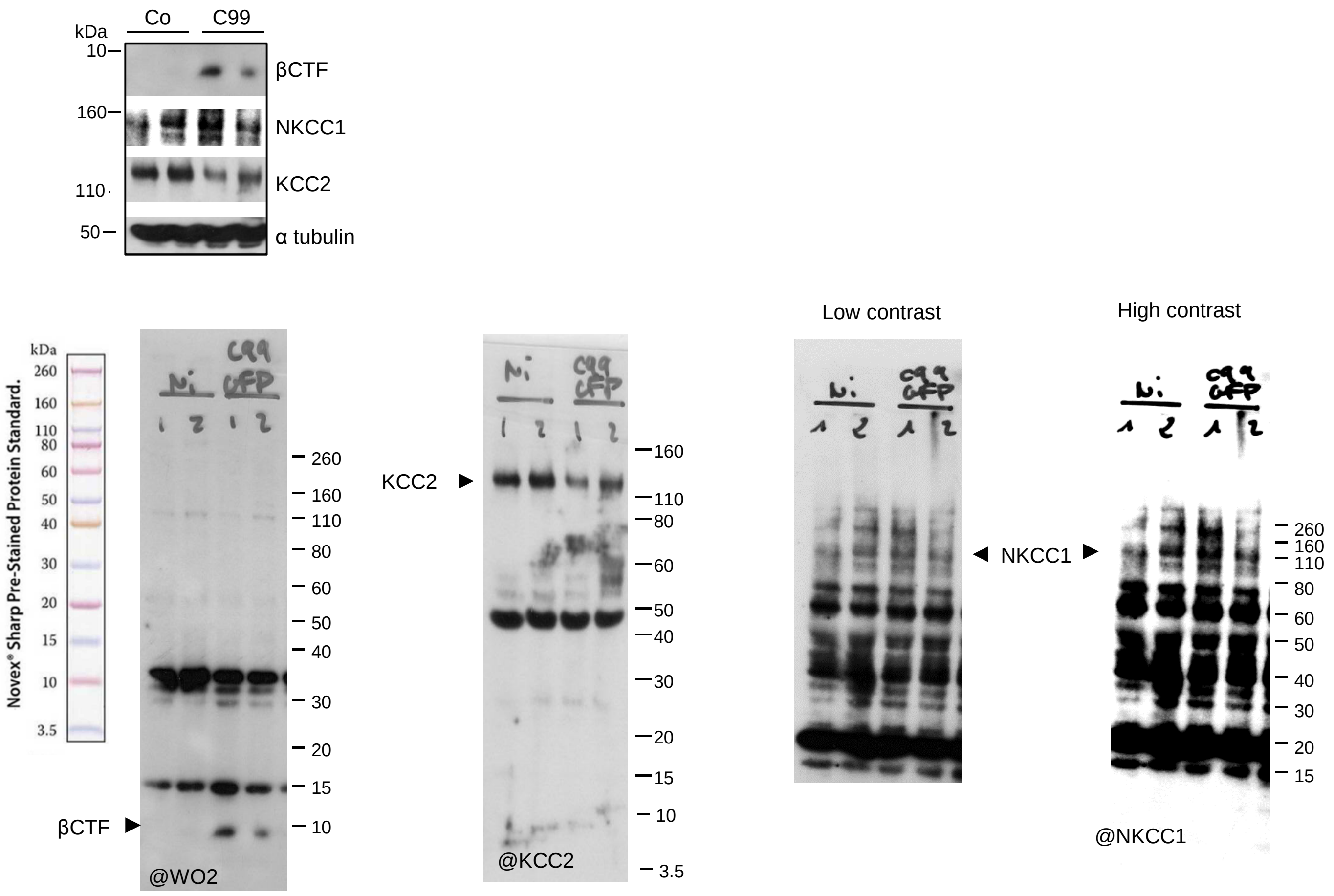

Fig. 3c

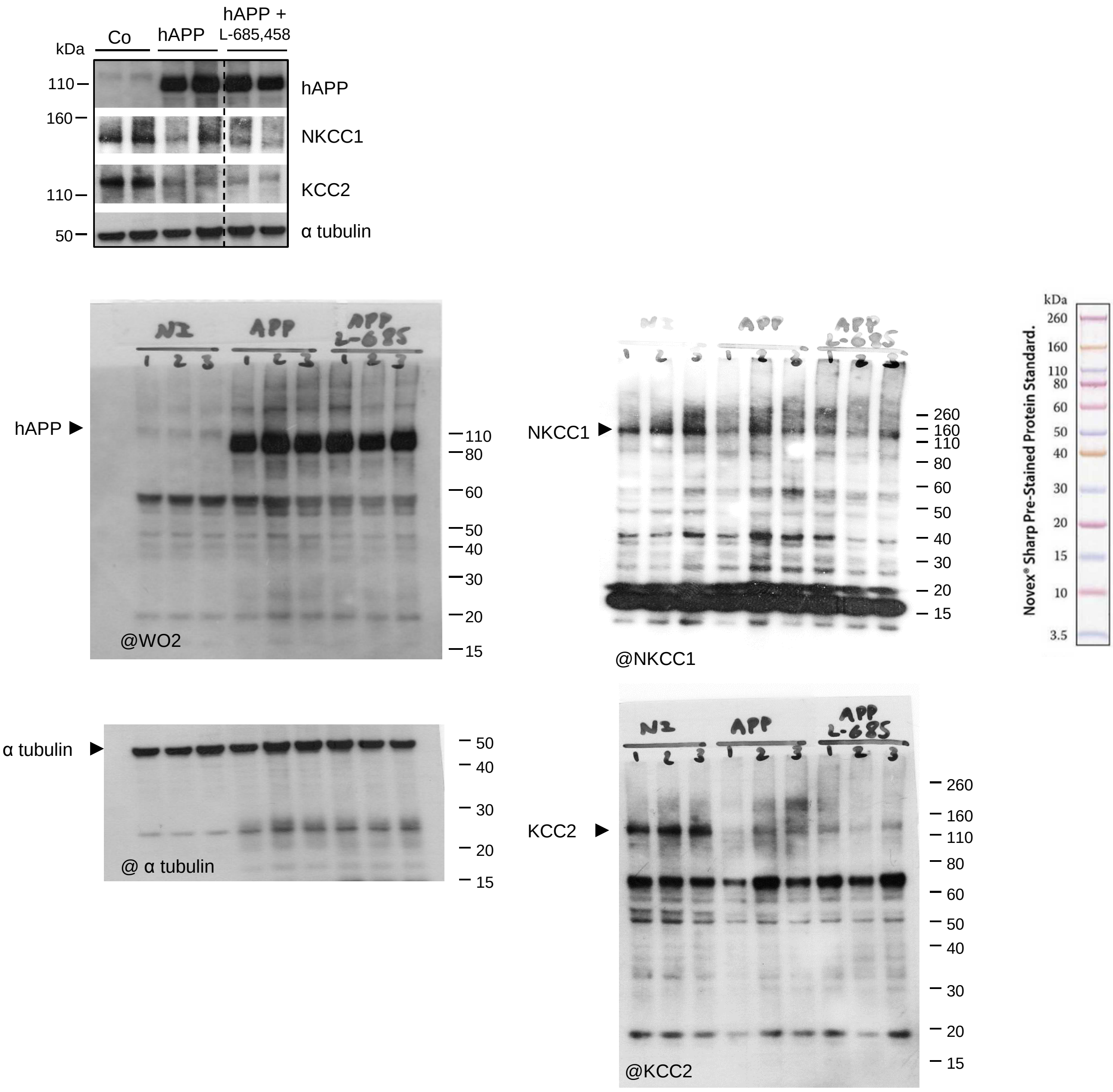

Co
hAPP
hAPP +
L-685,458
kDa
110
160
110
50
hAPP
NKCC1
KCC2
α tubulin
hAPP
110
80
60
50
40
30
20
15
@WO2
NKCC1
260
160
110
80
60
50
40
30
20
15
@NKCC1
kDa
260
160
110
80
60
50
40
30
20
15
10
3.5
Novex® Sharp Pre-Stained Protein Standard.
α tubulin
50
40
30
20
15
@ α tubulin
KCC2
260
160
110
80
60
50
40
30
20
15
@KCC2

Fig. 3e

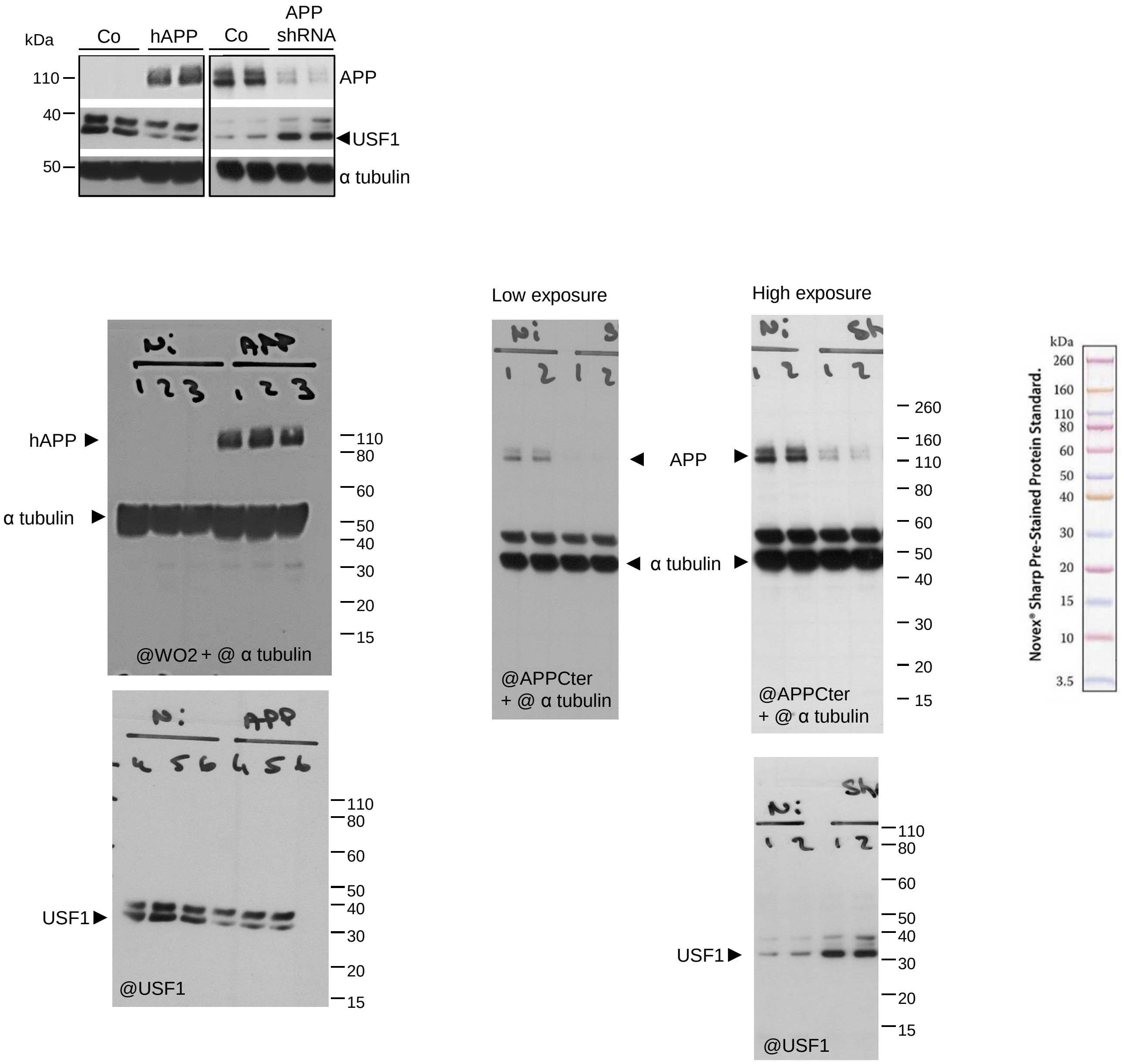
kDa
Co
hAPP
Co
APP shRNA
110
40
50
APP
USF1
α tubulin
hAPP
α tubulin
@WO2 + @ α tubulin
110
80
60
50
40
30
20
15
USF1
@USF1
Low exposure
High exposure
APP
α tubulin
@APPCter + @ α tubulin
260
160
@USF1
Novex® Sharp Pre-Stained Protein Standard.
3.5
10

Fig. 4a

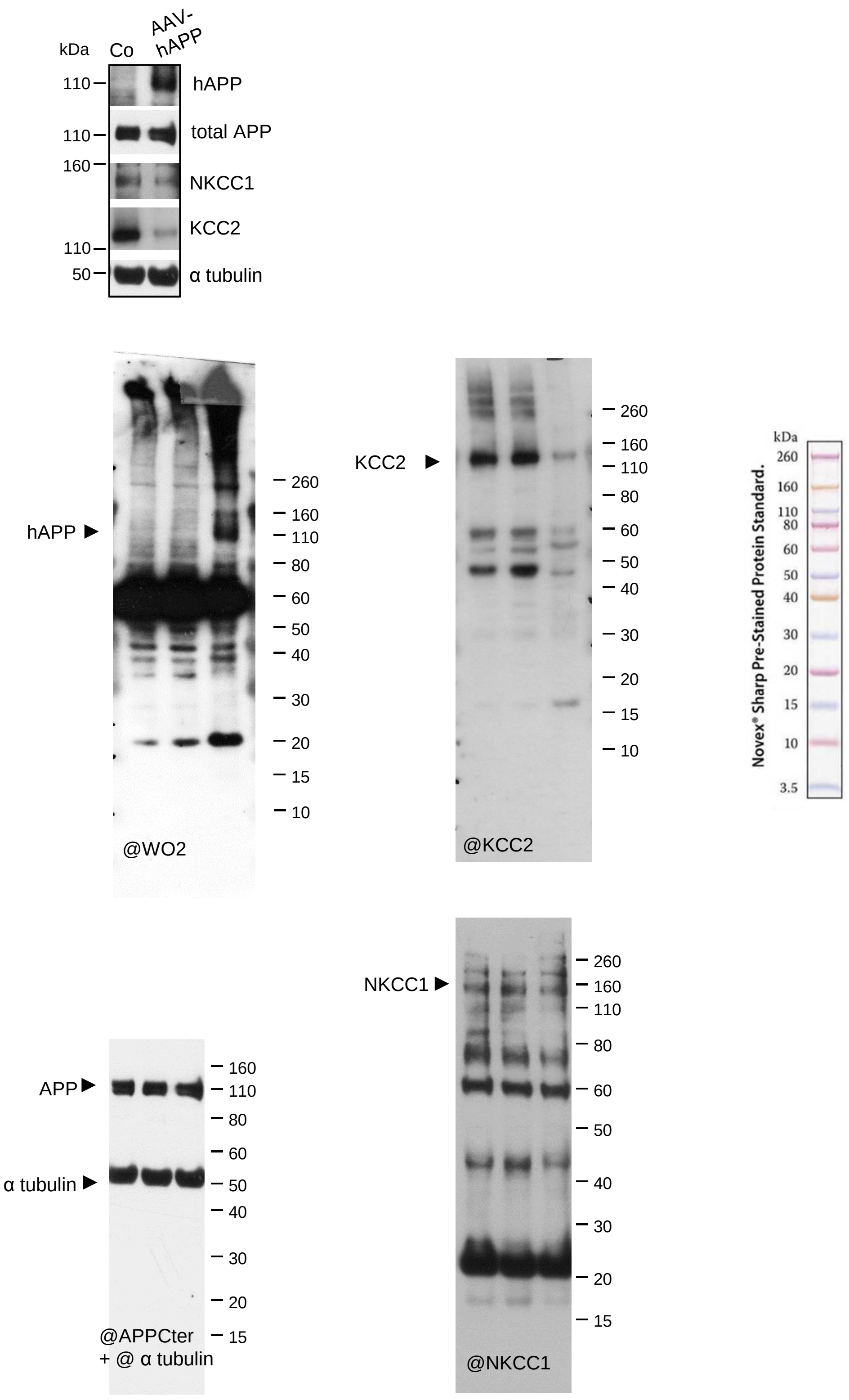

kDa
Co
AAV-hAPP
110
hAPP
110
total APP
160
NKCC1
KCC2
110
50
α tubulin
hAPP ▶
260
160
110
80
60
50
40
30
20
15
10
@WO2
KCC2 ▶
260
160
110
80
60
50
40
30
20
15
10
@KCC2
kDa
260
160
110
80
60
50
40
30
20
15
10
3.5
Novex® Sharp Pre-Stained Protein Standard.
APP ▶
α tubulin ▶
160
110
80
60
50
40
30
20
15
@APPCter
+ @ α tubulin
NKCC1 ▶
260
160
110
80
60
50
40
30
20
15
@NKCC1

Fig. 4b

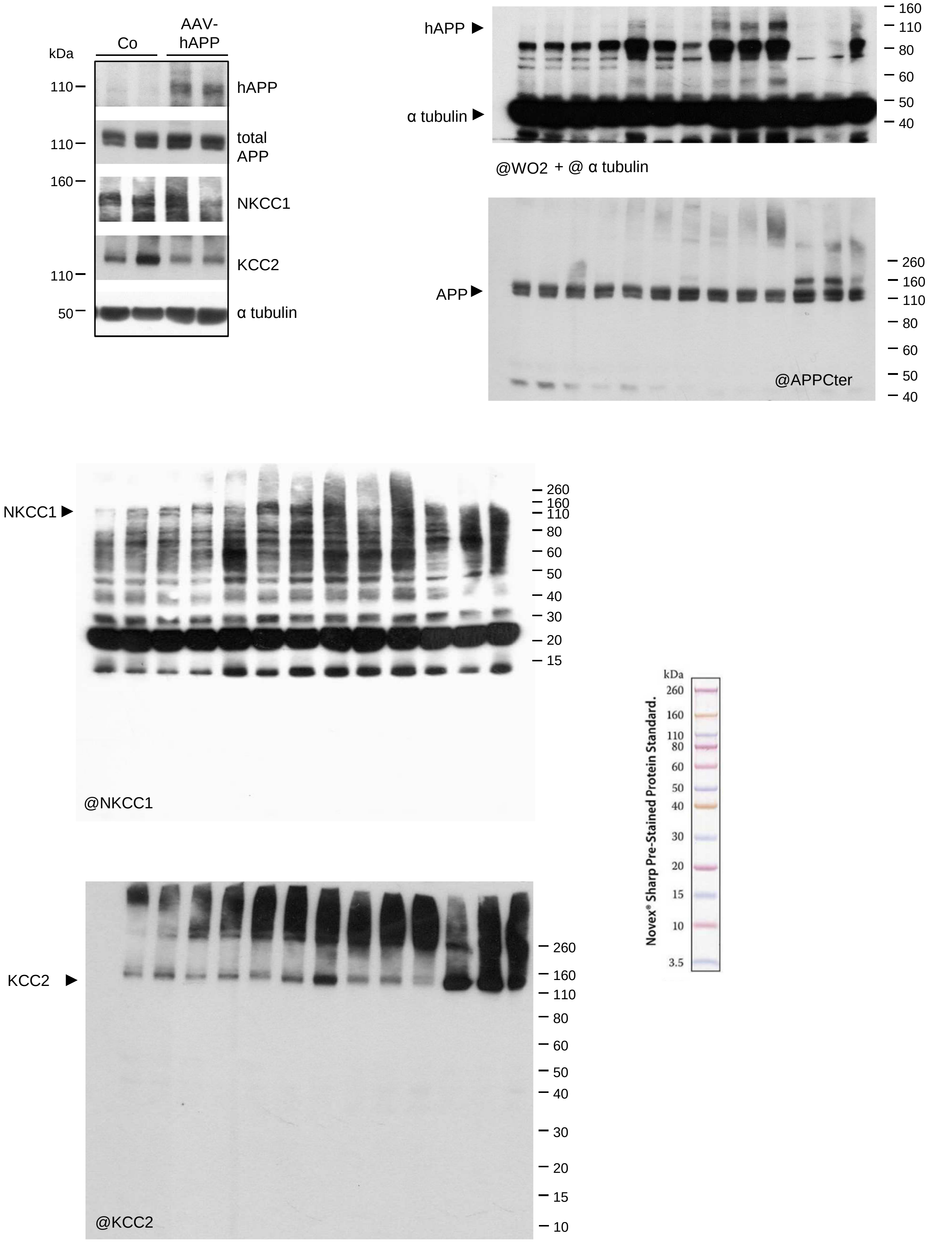
Co
AAV-hAPP
kDa
110
110
160
110
50
hAPP
total APP
NKCC1
KCC2
α tubulin
hAPP ▶
α tubulin ▶
160
110
80
60
50
40
@WO2 + @ α tubulin
APP ▶
260
160
110
80
60
50
40
@APPCter
NKCC1 ▶
260
160
110
80
60
50
40
30
20
15
@NKCC1
KCC2 ▶
260
160
110
80
60
50
40
30
20
15
10
@KCC2
Novex® Sharp Pre-Stained Protein Standard.
kDa
260
160
110
80
60
50
40
30
20
15
10
3.5
